# Knowledge management tools and mechanisms for evidence-informed decision-making in the WHO European Region: a scoping review

**DOI:** 10.1186/s12961-023-01058-7

**Published:** 2023-10-31

**Authors:** Fadi El-Jardali, Lama Bou-Karroum, Nadeen Hilal, Maya Hammoud, Nour Hemadi, Michelle Assal, Nour Kalach, Aya Harb, Natasha Azzopardi-Muscat, Tyrone Reden Sy, David Novillo-Ortiz

**Affiliations:** 1 Department of Health Management and Policy, Faculty of Health Sciences, American University of Beirut, Beirut, Lebanon; 2 Knowledge to Policy Center, Faculty of Health Sciences, American University of Beirut, Beirut, Lebanon; 3 Department of Health Research Methods, Evidence, and Impact, McMaster University, Hamilton, ON Canada; 4 Division of Country Health Policies and Systems, WHO Regional Office for Europe, Copenhagen, Denmark

**Keywords:** Evidence-informed decision-making, Knowledge management, WHO European Region, Scoping review

## Abstract

**Background:**

Knowledge management (KM) emerged as a strategy to promote evidence-informed decision-making. This scoping review aims to map existing KM tools and mechanisms used to promote evidence-informed health decision-making in the WHO European Region and identify knowledge gaps.

**Methods:**

Following the Joanna Briggs Institute (JBI) guidance for conducting scoping reviews, we searched Medline, PubMed, EMBASE, the Cochrane library, and Open Grey. We conducted a descriptive analysis of the general characteristics of the included papers and conducted narrative analysis of the included studies and categorized studies according to KM type and phase.

**Results:**

Out of 9541 citations identified, we included 141 studies. The KM tools mostly assessed are evidence networks, surveillance tools, observatories, data platforms and registries, with most examining KM tools in high-income countries of the WHO European region. Findings suggest that KM tools can identify health problems, inform health planning and resource allocation, increase the use of evidence by policymakers and stimulate policy discussion.

**Conclusion:**

Policymakers and funding agencies are called to support capacity-building activities, and future studies to strengthen KM in the WHO European region particularly in Eastern Europe and Central Asia. An updated over-arching strategy to coordinate KM activities in the WHO European region will be useful in these efforts.

**Supplementary Information:**

The online version contains supplementary material available at 10.1186/s12961-023-01058-7.

## Introduction

There is increased awareness and need among policymakers on the use of the best available research evidence and data to guide public health and health systems decisions. Barriers to evidence-informed policymaking included the large volume of evidence available and poor access to research [1, 2]. Knowledge management (KM) emerged as a strategy to promote evidence-informed decision-making as it is considered a way to provide the right information, to the right person, at the right time [3]. It involves the use of the most effective ways to create, share, translate and apply knowledge (tacit and explicit) in order to create value and improve effectiveness, as well as the enabling culture, processes and tools needed to do so [4, 5]. KM tools and strategies are essential to ensure easy access to information, tailored and targeted knowledge, effective dissemination and sharing among knowledge users [6]. In 2005, the World Health Organization (WHO) launched its global KM and its operational plan with the aim of strengthening national health systems through better KM, establishing KM in public health, and enabling WHO to become a better learning organization [5, 7]. The importance of knowledge generation, translation and dissemination was emphasized in the WHO thirteenth general programme of work (GPW13) covering the period 2019–2025 [8].

KM is central to achievement of Sustainable Development Goals by bridging the know-do gap and strengthening health systems. Effective KM tools and mechanisms can strengthen national health information systems through reducing data collection burden and proper management and use of big data to complement traditional methods for timely measurement and monitoring of health status and health system performance [9].

The COVID-19 pandemic has proved more than ever the importance of KM. The European Program of Work 2020–2025 emphasized the critical need for countries to strengthen their health data and information systems to ensure that decisions are data driven and facilitate public health monitoring [10]. In times of crisis, decisions are critical and the effectiveness of these decisions depends on effective KM systems which is the capacity to create, share, collect, transfer, and elaborate knowledge [11].

To our knowledge, there is no previous work that mapped KM initiatives, tools and mechanisms in the WHO European Region. This scoping review aims to map, identify knowledge gaps and provide an overview of available research evidence on existing KM tools and mechanisms used to promote evidence-informed decision-making in the WHO European Region. It also aims to examine implementation considerations and reported outcomes of the identified KM tools and mechanisms in public health, specifically health systems, in terms of promoting evidence-informed decision-making.

## Materials and methods

### Protocol and registration

We registered the protocol for this scoping review in Open Science Framework https://doi.org/10.17605/OSF.IO/Q2GTU.

### Definitions

A scoping review is typically used to present “a broad overview of the evidence pertaining to a topic, irrespective of study quality, to examine areas that are emerging, to clarify key concepts and to identify gaps”. We used the updated Joanna Briggs Institute (JBI) guidance for conducting scoping reviews [12]. We also followed the PRISMA Extension for Scoping Reviews (PRISMA-ScR) for reporting scoping reviews [13].

### Eligibility criteria

We included studies on KM tools and mechanisms based on traditional and digital data sources (e.g. communities of practice, networks, online registries, portals, information repositories, clinical guidelines or best practices, discussion forums, social media, electronic libraries, policy briefs). KM involves [14]:

- knowledge generation (knowledge acquisition, creation),
- knowledge storage (knowledge assimilation, package, documentation),
- knowledge processing (knowledge synthesis, integration, refinement),
- knowledge transfer (knowledge sharing, exchange, dissemination, brokering and translation),
- knowledge utilization.

We included studies that assess, examine or describe the role or the impact of the knowledge management tools and mechanisms on health policies and decision-making. We considered public policy that is any statement or position taken by the government or government departments. We excluded studies on knowledge management tools in clinical setting or health business or implemented at organizational level. We included primary studies, narrative reviews, systematic reviews, editorials and commentaries. We restricted our eligibility criteria to articles and reports published after the year 2005. We excluded protocols and abstracts of meetings and conferences. We restricted to studies focusing on the WHO European region (see Additional file 1: Appendix 1).

### Literature search

We searched the following electronic databases: Ovid Medline, PubMed, EMBASE, the Cochrane library, and Open Grey. We used both index terms and free text words for the three following concepts: knowledge management, policy and Europe. The search terms and MeSH terms for each database were developed with the guidance of an information specialist and with input from experts in KM. We also mapped studies and report on KM to identify additional search terms. We did not limit the search to specific languages. For articles in languages different than English, we used DeepL Translator (https://www.deepl.com/translator) to translate articles to English language. We ran the search from January 2005 till September 2022. We chose to restrict our search to 2005 as this year marks the rise of the “web 2.0” which had major implications on the internet in general and on knowledge management [15]. Search strategies are found in Additional file 2: Appendix 2.

### Selection process

We imported the results into Covidence (https://www.covidence.org/) where we conducted the selection process in two stages. Teams of two reviewers used the above eligibility criteria to screen titles and abstracts of identified citations in duplicate and independently for potential eligibility. We retrieved the full text for citations judged as potentially eligible by at least one of the two reviewers. Same teams of reviewers screened the full texts in duplicate and independently and resolved disagreements by discussion or with the help of a third reviewer. We pilot tested screening forms and conducted calibration exercises with a subset of studies to ensure the eligibility criteria are clear and reviewers are on high-level of agreement in the selection process.

### Data charting and synthesis

One reviewer abstracted data using standardized and pilot tested forms and another reviewer validated the extraction. The reviewers resolved any disagreement by discussion and when needed with the help of a third reviewer. We conducted pilot testing of the data extraction form to ensure the clarity and validity of the data abstraction process.

We extracted from each paper information on first authors (e.g. name and country of affiliation), year, language and type of publication, study design, setting (e.g. country(ies) subject of the paper and income level classification according to the World Bank list of economies issued in June 2021), characteristics of the intervention (type of KM tools/mechanisms, details, geographical/jurisdictional level, phase of KM (knowledge generation, storage, processing, transfer and utilization), key results, policy or decision examined (e.g. policies such as pharmaceutical policies, strategies, national health plans, national programs), statements on funding and conflict of interest of authors.

We conducted descriptive analysis of the general characteristics of the included papers including intervention, study designs, settings and outcome. We also conducted narrative analysis of the included studies and categorized studies according to KM type and phase.

### Risk of bias assessment

We did not conduct risk of bias assessment and methodological assessment of the quality of evidence, which is consistent with the Joanna Briggs Institute guidance manual.

## Results

### Study selection

**Fig. 1:**
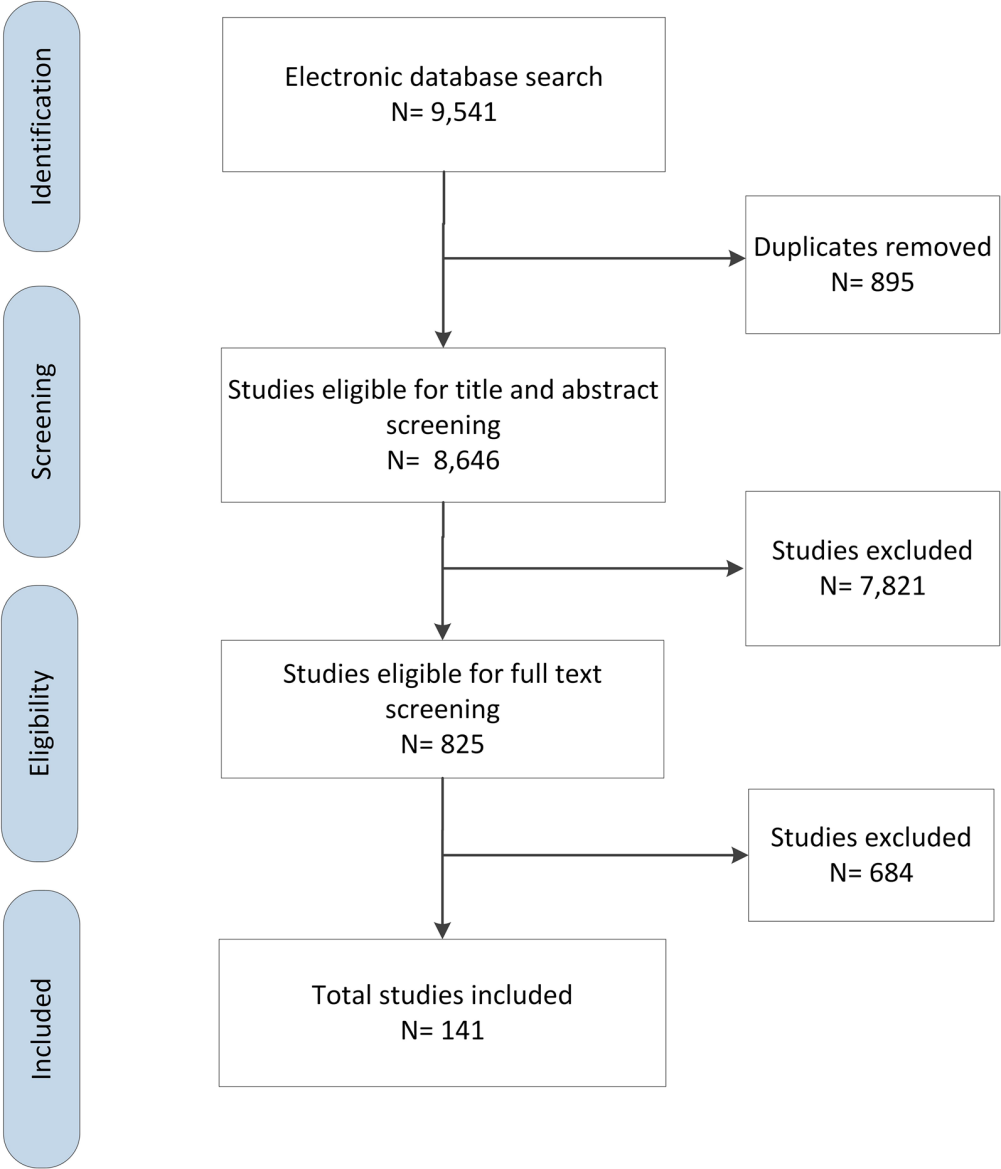
PRISMA flowchart

Figure 1 presents the PRISMA flowchart that summarizes the results of the search and selection process. Out of 9541 citations identified from electronic databases, we included 141 studies. At the full text screening, we excluded 684 articles for the following reasons: not outcome of interest (*n* = 324), not intervention of interest (*n* = 173), not design of interest (*n* = 104), missing full text (*n* = 48), not setting of interest (*n* = 31) and duplicate (*n* = 4).

### Characteristics of included studies

Table 1 presents the characteristics of included studies. Most of the studies examined KM tools in high-income countries of the WHO European region (*n* = 70; 49.6%) followed by studies examining knowledge management tools at a regional level or across different countries in the WHO European region (*n* = 68; 48.2%). Studies were mainly conducted by authors based in high-income European countries (*n* = 135; 95.7%). Many of the studies were descriptive case studies or employed observational study design. The KM tools mostly assessed in included studies were evidence networks and collaborations (*n* = 32; 22.7%) followed by surveillance tools, observatories, and data platforms (*n* = 23; 16.3%), and registries (*n* = 21; 14.9%). Most of the studies were reported as funded (*n* = 77; 51.8%) and reported no conflict of interest of authors (*n* = 73; 51.8%).

**Table 1:** Characteristics of included studies (*N* = 141)

	*N*	*%*
**Type of study design**		
Descriptive case studies	65	46.0
Observational studies (e.g. qualitative, surveys, Delphi techniques)	42	29.8
Literature/narrative reviews	9	5.6
Opinion pieces/editorials/commentaries	7	5.0
Technical reports	6	4.3
Systematic reviews	4	2.8
Quasi-experimental Studies	2	1.4
Policy briefs	2	1.4
Modelling studies	2	1.4
Book	1	0.7
Methodology paper	1	0.7
**Classification of the country of the institution to which the first author is affiliated**^a^		
European high-income countries	135	95.7
The United Kingdom	37	26.2
The Netherlands	18	12.8
Italy	12	8.5
Germany	12	8.5
Denmark	10	7.1
Belgium	10	7.1
Sweden	6	4.3
Hungary	4	2.8
Norway	4	2.8
Other European high-income countries	22	15.6
European upper middle-income countries	1	0.7
Bulgaria	1	0.7
European lower middle-income countries	0	0.0
European low-income countries	0	0.0
Non-European countries^b^	5	3.5
**KM phase and type**^c^		
Knowledge generation	11	7.8
Indicators	8	5.7
Surveys	3	2.1
Knowledge storage	48	34.0
Registries	21	14.9
Surveillance tools, observatories, and data platforms	23	16.3
Health information systems	4	2.8
Knowledge processing	24	17.0
Evidence synthesis	19	13.5
Health reports and toolkits	5	3.5
Knowledge transfer and utilization	59	41.8
Evidence networks and collaborations	32	22.7
Policy dialogues and stakeholders’ engagement	17	12.1
Community engagement	5	3.5
Decision support tools	5	3.5
**Country subject of intervention**^a^		
Regional level (i.e. WHO European Region)	68	48.2
European high-income countries	70	49.6
European upper middle-income countries	2	1.4
European lower middle-income countries	1	0.7
European low-income countries	0	0.0
**Reporting of funding**		
Reported as funded	77	54.6
Reported as not funded	12	8.5
Not reported	52	36.9
**Reporting of conflict of interest**		
Reported as no conflict of interest	73	51.8
Reported as conflict of interest	17	12.1
Not reported	51	36.2

^a^As per World Bank list of economies issued in June 2021

^b^Countries outside the WHO European region

^c^More than one option can apply

### Findings

**Fig. 2:**
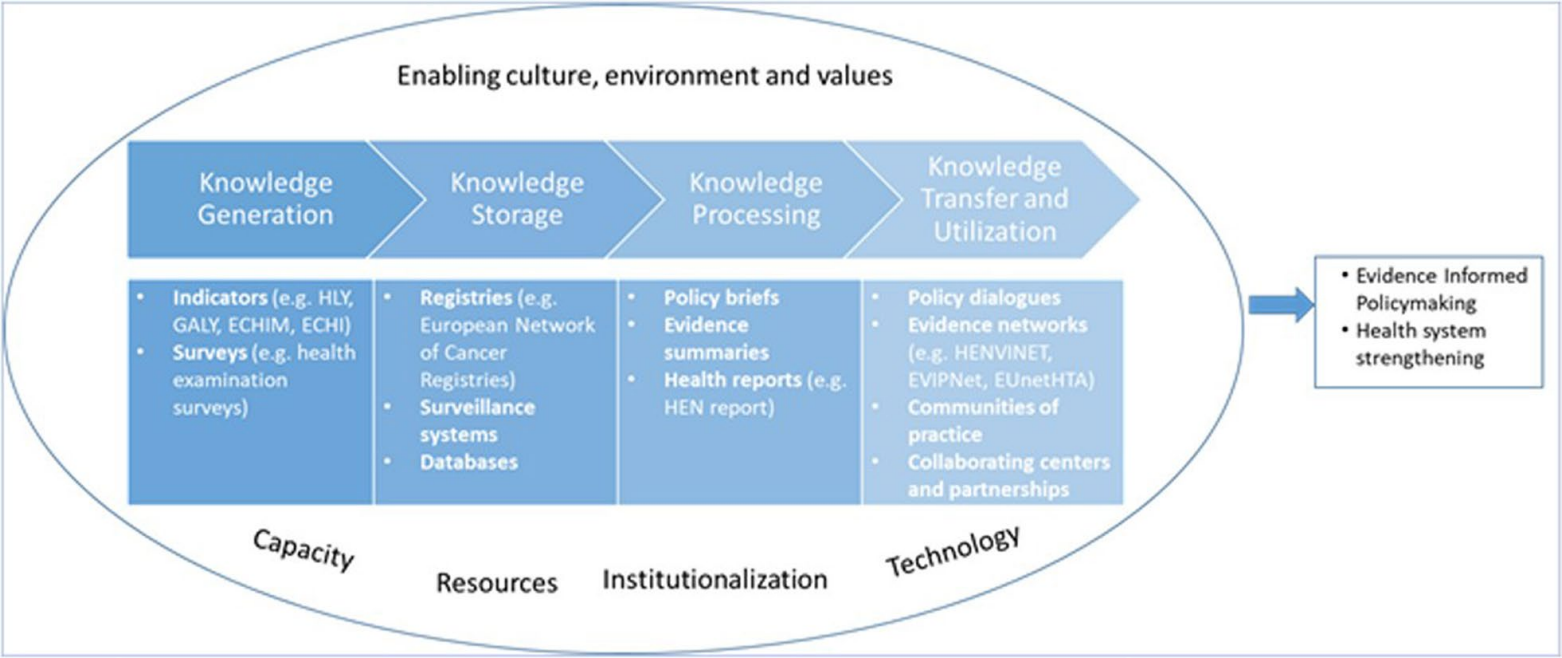
Summary of findings

Figure 2 summarizes the study findings briefly. We provide below the narrative analysis of the findings categorized by KM phase and type. We presented the implementation considerations including barriers and facilitators contributing to the successful implementation of the different KM tools in Table 2.

**Table 2:** Implementation considerations for different KM tools and mechanisms

	Facilitators/benefits	Major challenges/barriers
Knowledge generation	- Data availability [16, 18, 19] - Data completeness [16, 18, 19] - Up-to-date data [16, 18, 19] - Standardization and regularity of data collection and reporting mechanisms [16, 18, 19, 26] - Policy relevance [19] - Training [26]	- Missing data [16]
Knowledge storage	- Use of computerization [28] - Use of standard terminology - Better design of reporting systems [39] - Standardization and harmonization of data collected [39, 151] - Re-evaluation of the case definition [39] - Data completeness and accuracy [44] - Clear methodology for the development of a registry [34] - Availability of a central contact point [32] - Ability to share data with regulatory authorities [32] - Linkages to external databases [151] - Access to and ownership of original data [62] - Training of personnel and users [32] - Confidentiality of data [62] - Interactions with variety of stakeholders [62] - Political buy-in [32]	- Lack of mandatory notification requirement [39] - Lack of standardized definition for the disease [39] - Limited funding [62, 151] - Limited staff training [62] - Poor data quality [44]
Knowledge processing	- Applicability of the evidence to the context [77, 78] - Length and language of the summary [77, 78] - Standardized approach [72, 73] - Team with complementary skills and expertise [72, 73] - Expertise of external partners [72, 73] - Institutionalizing the use in decision-making [82]	- Difficulties of the statistical and scientific terms [77, 78]
Knowledge transfer	- Ensuring regularity of dialogues [103, 104] - Follow up with stakeholders afterwards [103, 104] - Applicability of evidence [114] - Availability of relevant data and research [114] - Improved dissemination and access to research [114, 130] - Administrative support [114] - Training of personnel [114] - Research co-production [114, 130] - Joint knowledge agenda [131] - Research-led by people embedded in the contexts in which the results can be used [114, 130] - Recognizing the role of contextual factors [105, 106] - Availability of resources [105, 106]	- Time constraints [114, 115] - Lack of funding and limited resources [114, 115] - Lack of priority on the policy agenda [114, 115] - Limited availability of data [152] - Uncertainty about potential data sources [152] - Lack of skilled policymakers [114] - Insufficient institutional research capacity [114] - Opposing interests [115]

### Knowledge generation

#### Indicators (*n* = 8)

Eight studies assessed the role of indicators in evidence-informed policymaking (Additional file 3: Appendix 3). The indicators examined in the studies are EURO-HEALTHY PHI [16], HLY—a disability-free life expectancy, the GALI [17], ECHIM [18], ECHI [19], measurable indicators for evidence-informed policy-making developed by REPOPA project [20], key performance indicators (KPIs) in regional-level health-care systems [21]. Indicators such as HLY, DALY and GALI indicators were used to set policy targets, develop strategies in health such as national health plans and design policies and programs and evaluate national programs and service provision [17, 22, 23].

#### Surveys (*n* = 3)

Three studies assessed the use of surveys and randomized controlled trials in generating knowledge to inform policy and health planning [24–26] (Additional file 4: Appendix 4). The European health examination surveys (HES) have the potential to identify priorities health problems to be addressed and can be used for health monitoring.

### Knowledge storage

#### Registries (*n* = 21)

Twenty-one studies focused on the role of registries in decision-making (Additional file 5: Appendix 5). Cancer registries, at the national and regional levels, received special attention among registries targeting specific diseases and were found to help establish public health priorities, guide resource allocation, inform decisions regarding reimbursement, access and care delivery and support planning and evaluation of health services [27–31].

Other registries included rare disease registries [32–36], registries addressing neurological and neurodevelopmental diseases such as multiple sclerosis [37] and autism [38] and registries on infectious diseases [39] and non-communicable diseases [40–42]. These registries enabled health authorities and policymakers to identify at-risk groups for which targeted care is needed and to develop programs responsive to the patients’ needs and supports the planning and implementation of public health policies toward disease management and control. Aside from the role assumed by registries in disease management and service delivery, population registries can support governmental and authoritative decisions such as planning and resource allocation and measures such as taxation, allowance, and subsidies [43]. Data generated through registries can also be used for the regulation of medical supplies and the medical profession [44, 45].

### Surveillance tools, observatories, and data platforms (*n* = 24)

Twenty-four studies on surveillance, observatories, and data platforms were included in the review (Additional file 6: Appendix 6). Surveillance systems were an integral part of the response to the COVID-19 pandemic in Denmark and Italy through guiding the national policies [46] and analyzing the pandemic evolution [47]. Similarly, the West Nile virus surveillance system in Italy and the Portuguese Tuberculosis Surveillance System were developed to guide public health policies designed to mitigate the risk of disease transmission [48, 49]. Health surveillance systems in Germany of emergency admissions enables continuous monitoring of relevant health phenomena issues thus can guide evidence-informed decision-making [50, 51].

Three studies found that observatories can monitor health systems performance [52], provide policy options for the development of several health-related policies such as funding of long-term care and anti-tobacco policies, through packaging and sharing information with policymakers [53] and promote common methods for responding to global eHealth challenges [54].

Data platforms can also support policy and decision-making on drug regulation [55] and other health issues such as childhood obesity, tobacco, nutrition and COVID-19 response. Examples of data platforms addressing nutrition issues included the food composition data in the European region [56], and the Nutri-RecQuest, a regional data platform in the EU [57]. Other data platforms included the BigO tool in Greece, Sweden, and Ireland [58], European Health Information Gateway [59], the Big Data platform [60, 61], the Climate-Environment-Health data mashup [62], the ADR NI Database [63], the Atlas of Cardiology [64], the web portals deployed during COVID-19 [65], EUPHIX [66], e-labs [67] and the European Service Mapping Schedule/Description and Evaluation of Services and DirectoriEs system [68].

#### Health information systems (*n* = 4)

Four studies discussed the essential role of health information systems in the EU in providing the base for health planning and policymaking (Additional file 7: Appendix 7). All of these studies discussed health information systems at the regional level of the EU. These studies highlighted the fragmentation and diversity of health information systems across EU and the need to harmonize and standardize and ensure systematic data collection and reporting [69–71] and the need to leverage on digital health [65]. One study discussed the need to integrate information on refugees and migrants within the health information systems in the EU to allow for better health planning [69].

### Knowledge processing

#### Evidence synthesis (*n* = 19)

Nineteen studies examined the role of evidence synthesis in informing policies and decisions in Europe (Additional file 8: Appendix 8). Evidence briefs for policy, evidence guides, context-specific evidence summaries, scoping and rapid reviews and plain language summaries of systematic reviews can play a role in informing strategies, plans and decisions [72–76] and considered as a credible and useful source of information [77, 78]. Demand-led evidence briefing service, a resource-intensive service, was not associated with increases in NHS commissioners capacity to acquire, assess, adapt and apply research evidence to support decision-making compared with less intensive and less targeted strategies [79]. Included studies showed that HTA [80–83], CED schemes [84, 85], evidence-based national guidelines [86] and DECIDE tool [87] can inform resource allocation and reimbursement decisions to create the most value for money. Data mining and public health triangulation was also identified as tools to support decision-making in public health [88, 89].

#### Health reports and toolkits (*n* = 5)

Five studies examined how health reports and toolkits can support in monitoring health systems, developing and implementing national policies and influencing decision-making process [90–93] (Additional file 9: Appendix 9). The health reports and toolkits identified in the included studies were the WHO HEN reports and the European Health Report published at the WHO Europe level [59, 91], the health care performance report [90], the Public Health Status and Foresight report published in the Netherlands [92] and the Healthy Eating and Physical Activity in Schools toolkit [93].

### Knowledge transfer

#### Policy dialogue and stakeholder involvement (*n* = 17)

Seventeen studies examined how policy dialogues and stakeholder involvement can inform decision-making (Additional file 10: Appendix 10). Policy dialogues and stakeholder involvement, at the national and sub-national levels, can increase the use of research evidence by policymakers, increased policymaker’s awareness, facilitated interaction between a range of stakeholders across different sectors, provided conducive environment for discussion of timely and relevant summarized evidence and led to adoption, development and changes in policies and strategies [94–110]. Included studies reported that the dialogues were informed by evidence such as HTA, systematic reviews and context-specific reports.

#### Evidence networks and collaborations (*n* = 32)

Evidence networks and collaborations promote partnerships between key stakeholders including policymakers, researchers, and academic bodies to inform public policy (Additional file 11: Appendix 11). Evidence networks across Europe such as the HENVINET [111, 112], the HEN [113], EVIPNet [114, 115], Burden-eu [116], EurOOHnet [117], the European network on human biomonitoring [118], the HBM4EU and BRIDGE health [119] and other stakeholders networks and knowledge brokering activities [120–122] are able to support decision makers across key public health issues such as context-specific diet and nutrition policies [113] and pharmaceutical policies [123]. The EUnetHTA, HTAB and epistemic communities also facilitated linking the HTA evidence to policymaking [123–127].

National evidence networks such as the Finnish National Healthy Cities Network, the Knowledge Transfer Partnership in Scotland, the Share-Net in the Netherlands and Life Science Exchange project contributed to the development, implementation and evaluation of health policies and services [128–131]. Other national evidence networks and expert committees provided policy advice during the COVID-19 pandemic [132–134]. Collaborations at the research and academic levels act as KM tools and form an evidence base for public health policy and practice [135, 136]. AskFuse, a knowledge brokering service provided a platform for collaboration between researchers and policymakers [137]. Two studies reported on policy games simulations, bringing policymakers together to jointly develop a policy implementation plan [138, 139].

#### Community engagement (*n* = 5)

Five studies examined the influence of community engagement on decision-making (Additional file 12: Appendix 12). Community engagement can provide evidence to policymakers to ensure health reforms included a focus on social determinants of health [140], ensure health services are designed to meet the needs of the targeted population [141, 142], refine service delivery [143] and inform national policies on controlling alcohol availability [144].

#### Decision support tools (*n* = 5)

DSTs can play an important role in transferring information and knowledge to policy and decision makers on road safety, health services, environmental and urban health [145–149] (Additional file 13: Appendix 13). DSTs assessed in the included studies were the CRAFT tool [148], the HENVINET DST MDB [150], the NHS Scotland DST Platform [148], the SOMNet, combined with the EbCA [149] and the European Road Safety Decision Support System [147].

## Discussion

This scoping review maps the evidence on KM tools and mechanisms aiming at influencing policy decisions-making and promoting evidence-informed decision-making in the WHO European Region. It identifies 141 studies assessing different KM tools and mechanisms. Findings suggest that knowledge management tools can identify health problems, inform health planning and resource allocation and can be used for health monitoring. Most of the included studies stressed on the importance of the availability of resources, the sustainability and the institutionalization of the use of KM tools and mechanisms in order to promote the use of evidence and knowledge generated in decision making. Political commitment and creating the adequate culture are essential to increase the uptake of evidence generated from different KM tools and mechanisms.

The KM tools mostly assessed were evidence networks and collaborations, surveillance tools, observatories, and data platforms and registries. The majority of the studies examined knowledge management tools implemented in high-income countries of the WHO European region. This finding can be interpreted by the fact that research and work on KM in other parts of the WHO European Region is still in its earliest phase. It can also be explained by the limited resources available in these countries to invest in KM.

Many studies examined KM tools at a regional level, which shows initiatives at the WHO European region level to invest and advance the work on KM. This finding is validated by the range of evidence networks and collaborations that was identified in this review such as HENVINET, EVIPNet, EUnetHTA, HBM4EU and BRIDGE Health. The majority of the included studies were conducted by authors based in high-income Europe. This finding shows the imbalance in research capacities between high-income and low and middle-income countries in the WHO European region.

The majority of the studies employed descriptive case study or observational designs as opposed to experimental studies. This can be interpreted by the difficulty of applying experimental design and the multiple and complex factors that affect the policymaking process which make it hard to evaluate the direct impact of KM tools and mechanisms on decision-making.

Ensuring data quality, harmonization and completeness and regularity of data collection was reported as a key factor for the success of health information systems, registries, surveillance tools, observatories, and data platforms. These pillars would allow comparability of data across countries across the WHO European Region and over time. Integrating all sections of the population such as refugees, migrants, and other marginalized or disadvantaged population was reported to be essential for better health planning [153]. These findings call for supporting work in Central Asia (CA) and Eastern Europe (EE) in data harmonization and completeness as part of health information systems strengthening outlined in the EPW and GPW13 and as a catalyst in the development of KM platforms and tools.

Plain language summaries of systematic reviews, evidence briefing services, scoping and rapid reviews were found to be useful sources of information for policymakers. Researchers and institutions working in developing those summaries should take into consideration the applicability of the evidence to the context, the difficulties of the statistical and scientific terms, the length of the summary and the language. Evidence synthesis was shown to support decision-making in other regions [154] and mainly during COVID-19 [155].

Evidence networks and collaborations across Europe were also found to support decision-makers across key public health issues. These evidence networks were also shown to support decision-making in other jurisdictions such as the Americas [156]. Policy dialogues were shown to increase the use of research evidence by policymakers, increased policymaker’s awareness and stimulate discussion on the issue raised during the dialogue and facilitated the interaction between a range of stakeholders across different sectors [157, 158]. To ensure desired impact from the dialogues, there is a need to conduct periodic dialogues, follow up with stakeholders afterwards and recognize the role of contextual factors and ensure availability of resources for implementation. In addition to engaging stakeholders, engaging communities is essential to include the voice of citizens in policymaking. However, most of the studies on policy dialogues showed that these dialogues are conducted mainly at national levels as opposed to conducting them at a regional level (i.e. WHO European Region).

## Strengths and limitations

To our knowledge, this is the first study to map the published evidence on KM tools and mechanisms aiming at influencing decision making in the WHO European Region. One strength of the study is that we followed Joanna Briggs Institute (JBI) guidance for conducting scoping reviews [12] and we followed the PRISMA Extension for Scoping Reviews (PRISMA-ScR) for reporting scoping reviews [13]. Our scoping review has three main limitations. The first limitation is that we did not search Russian-language scientific databases so we might have missed studies conducted in Russian-speaking countries. Second limitation is that the framework we used consider knowledge translation as part of knowledge management. While we consider the knowledge translation as a sub-set of knowledge management, we acknowledge the distinct focused, scopes and processes of knowledge translation within the larger framework of knowledge management. Third, we acknowledge that our search strategy might have missed certain types of KM tools such as the Evidence to Decision (EtD) framework due to the restriction to certain names of KM tools in the search strategy.

## Implications for research and policy

This scoping review can inform researchers and funders interested in understanding the role of KM tools and mechanisms in influencing health decision-making mainly in the WHO European region. While we acknowledge the challenges of measuring the effectiveness of knowledge management tools on decision making, researchers are encouraged to conduct better-designed and rigorous research studies to assess this relationship to inform efforts aiming at promoting evidence-informed decision-making in this region mainly in CA and EE countries. Researchers are also called to develop and follow guidelines for designing and reporting studies evaluating the effectiveness or impact of KM tools and mechanisms. Our scoping review can also inform the work researchers aiming at mapping KM initiatives, tools and mechanisms in other WHO regions.

As plain language summaries, policy dialogues and evidence networks were shown to increase the use of research evidence by policymakers and stimulate discussion on policy issues, funders are called to support capacity-building activities in this aspect, particularly in the eastern part of the WHO European Region, where research production and KM activities are still at their early stages. Given that most studies on KM systems, tools, and platforms found were from high-income countries in Western Europe, there is a need for further understanding the needs of the CA and EE countries for KM platforms and systems, and accordingly conduct twinning and knowledge exchange activities between high income countries with developed KM systems and platforms with countries who still lag behind. The findings also highlight the need to institutionalize the use of evidence in decision-making and leverage on existing KM tools and mechanism to inform health policies and national strategies.

Health systems managers and policymakers are called to ensure data availability, completeness, and standardization of data collection and reporting mechanisms to improve their country’s health information systems and the work of registries, surveillance tools and observatories. These KM tools would allow for better health planning including resource allocation and reimbursement decisions.

### Supplementary Information

**Additional file 1: Appendix 1: **List of Member States of the WHO European Region.

**Additional file 2: Appendix 2: **Search strategies.

**Additional file 3: Appendix 3.** Table of characteristics - Indicators.

**Additional file 4: Appendix 4.** Table of characteristics - Surveys.

**Additional file 5: Appendix 5.** Table of characteristics - Registries.

**Additional file 6: Appendix 6.** Table of characteristics - Surveillance and Observatories.

**Additional file 7: Appendix 7.** Table of characteristics - HIS.

**Additional file 8: Appendix 8.** Table of characteristics - Evidence Synthesis.

**Additional file 9: Appendix 9.** Table of characteristics - Health Reports.

**Additional file 10: Appendix 10.** Table of characteristics - Policy dialogues.

**Additional file 11: Appendix 11.** Table of characteristics - Evidence networks.

**Additional file 12: Appendix 12.** Table of characteristics - Community engagement.

**Additional file 13: Appendix 13.** Table of characteristics - Decision Support Tools.

## Data Availability

The datasets supporting the conclusions of this article are included within the article and its Additional files.

## Abbreviations

DSTs: Decision support tools
HENVINET DST MDB: Health and Environment Network Decision Support Tools Metadata Base
CRAFT: Cities Rapid Assessment Framework for Transformation
GIFT: Global Individual Food consumption data Tool
ADR NI: Administrative Data Research Northern Ireland
EUPHIX: European Public Health Information and Knowledge System
WHO: World Health Organization
HEN: Health Evidence Network
HTA: Health Technology Assessment
CED: Coverage with Evidence Development
Burden-eu: European Burden of Disease Network
HBM4EU: European human biomonitoring initiative
ECHI: European Core Health Indicators
PHI: Population Health Index
ECHIM: European Community Health Indicators & Monitoring
REPOPA: REsearch into POlicy to enhance Physical Activity
GALI: Global Activity Limitation Indicator
SOMNet: Self-organising map network
EbCA: Expert-based collaborative analysis
HLY: Healthy Life Years
HENVINET: Health and Environment Network
