# Supplementary material for: Knowledge management tools and mechanisms for evidence-informed decision-making in the WHO European Region: a scoping review
Source: Health Res Policy Syst. 2023 Oct 31;21:113. doi: 10.1186/s12961-023-01058-7 (PMC10619313; doi:10.1186/s12961-023-01058-7)
Supplement: Supplementary file 1 — Additional file 1: Appendix 1: List of Member States of the WHO European Region. [file 12961_2023_1058_MOESM1_ESM.docx]

# Appendix 1: List of Member States of the WHO European Region

| Albania | Greece | Poland |
| --- | --- | --- |
| Andorra | Hungary | Portugal |
| Armenia | Iceland | Republic of Moldova |
| Austria | Ireland | Romania |
| Azerbaijan | Israel | Russian Federation |
| Belarus | Italy | San Marino |
| Belgium | Kazakhstan | Serbia |
| Bosnia and Herzegovina | Kosovo^[[1]](#footnote-1)^ | Slovakia |
| Bulgaria | Kyrgyzstan | Slovenia |
| Croatia | Latvia | Spain |
| Cyprus | Lithuania | Sweden |
| Czech Republic | Luxembourg | Switzerland |
| Denmark | Malta | Tajikistan |
| Estonia | Monaco | Türkiye |
| Finland | Montenegro | Turkmenistan |
| France | Netherlands | Ukraine |
| Georgia | North Macedonia | United Kingdom of Great Britain and Northern Ireland |
| Germany | Norway | Uzbekistan |

1. All references to Kosovo in this document should be understood to be in the context of United Nations Security Council Resolution 1244 (1999) [↑](#footnote-ref-1)
