# Supplementary material for: Knowledge management tools and mechanisms for evidence-informed decision-making in the WHO European Region: a scoping review
Source: Health Res Policy Syst. 2023 Oct 31;21:113. doi: 10.1186/s12961-023-01058-7 (PMC10619313; doi:10.1186/s12961-023-01058-7)
Supplement: Supplementary file 2 — Additional file 2: Appendix 2: Search strategies. [file 12961_2023_1058_MOESM2_ESM.docx]

# Appendix 2: Search strategies

## Medline search strategy

Database: Ovid MEDLINE(R) and Epub Ahead of Print, In-Process, In-Data-Review & Other Non-Indexed Citations and Daily <1946 to October 17, 2022>

Search Strategy:

--------------------------------------------------------------------------------

1 ((knowledge or evidence or information or data) adj2 (management or creation or storage or storing or exchang* or sharing or transfer* or translation or generation or generating or mobili?ation or integration or application or brokering or dissemination or acquisition)).tw. (132912)

2 ((knowledge or research or information) adj2 (network* or platform or repository or database)).tw. (34080)

3 (communit* adj2 practice).tw. (6215)

4 Translational Medical Research/ (12733)

5 Knowledge Management/ (399)

6 ((reporting or tracking) adj2 system?).ti,ab. (18987)

7 ((evidence or policy) adj2 brief?).ti,ab. (912)

8 ((deliberative or policy) adj2 dialogue?).ti,ab. (332)

9 (registry or registries or repositories or repository or portals or e-libraries).ti,ab. (186246)

10 (discussion adj forum?).ti,ab. (952)

11 (Electronic adj libraries).ti,ab. (47)

12 Social Networking/ (5127)

13 (blog* or microblog* or weblog*).ti,ab. (2718)

14 or/1-13 (387857)

15 Public Policy/ or policy making/ or health policy/ or health systems plans/ or health planning/ (133610)

16 Decision Making/ (103364)

17 (policymaking or policy-making or policy-maker? or decision-maker? or decision-making or "decision making").ti,ab. (220004)

18 ((policy or decision?) adj (maker? or making)).ti,ab. (218069)

19 (evidence adj (based or informed) adj (practice or policy or policies or program? or decision?)).tw. (16793)

20 or/15-19 (413215)

21 europe/ or andorra/ or austria/ or balkan peninsula/ or belgium/ or europe, eastern/ or albania/ or baltic states/ or estonia/ or latvia/ or lithuania/ or "bosnia and herzegovina"/ or bulgaria/ or croatia/ or czech republic/ or hungary/ or kosovo/ or "republic of north macedonia"/ or moldova/ or montenegro/ or poland/ or "republic of belarus"/ or romania/ or russia/ or serbia/ or slovakia/ or slovenia/ or ukraine/ or france/ or germany/ or exp united kingdom/ or greece/ or ireland/ or italy/ or liechtenstein/ or luxembourg/ or monaco/ or netherlands/ or portugal/ or san marino/ or denmark/ or finland/ or iceland/ or norway/ or sweden/ or spain/ or switzerland/ (1451318)

22 (Europe* or Albania or Andorra or Armenia or Austria or Azerbaijan or Belarus or Belgium or "Bosnia and Herzegovina" or Bulgaria or Croatia or Cyprus or Czechia or Denmark or Estonia or Finland or France or Georgia or Germany or Greece or Hungary or Iceland or Ireland or Israel or Italy or Kazakhstan or Kosovo or Kyrgyzstan or Latvia or Lithuania or Luxembourg or Malta or Monaco or Montenegro or Netherlands or Macedonia or Norway or Poland or Portugal or Moldova or Romania or Russia? or "San Marino" or Serbia or Slovakia or Slovenia or Spain or Sweden or Switzerland or Tajikistan or Turkey or Turkmenistan or Ukraine or "United Kingdom" or Britain or Ireland or Uzbekistan).ti,ab. (1048567)

23 21 or 22 (2003408)

24 14 and 20 and 23 (2974)

25 limit 24 to ez="20210901-20220930" (248)

26 limit 24 to yr="2005 - 2021" (2435)

***************************

## PubMed search strategy

| Search number | Query | Sort By | Filters | Results | Time |
| --- | --- | --- | --- | --- | --- |
|  |  | | 2021/09:2022/09 [crdt] | 505 | 5:01:12 |
| 20 | #10 AND #17 AND #18 | | from 2005 - 2021 | 4,112 | 5:00:56 |
| 19 | #10 AND #17 AND #18 | | | 4,558 | 5:00:21 |
| 18 | #14 OR #15 | |  | 1,975,375 | 4:59:44 |
| 17 | #11 OR #13 OR #16 | |  | 765,084 | 4:58:43 |
| 16 | policymaking [TIAB] OR policy-making [TIAB] OR policy-maker [TIAB] OR policy-makers [TIAB] OR decision-maker [TIAB] OR decision-makers [TIAB] OR decision-making [TIAB] OR decision making [TIAB] OR decision-maker [TIAB] OR decision-makers [TIAB] OR decision maker [TIAB] OR decision makers [TIAB] OR policy [TIAB] OR policies [TIAB] OR decision [TIAB] OR decisions [TIAB] | | | 688,477 | 4:56:43 |
| 15 | Europe [TIAB] OR European [TIAB] OR Albania [TIAB] OR Andorra [TIAB] OR Armenia [TIAB] OR Austria [TIAB] OR Azerbaijan [TIAB] OR Belarus [TIAB] OR Belgium [TIAB] OR "Bosnia and Herzegovina" [TIAB] OR Bulgaria [TIAB] OR Croatia [TIAB] OR Cyprus [TIAB] OR Czechia [TIAB] OR Denmark [TIAB] OR Estonia [TIAB] OR Finland [TIAB] OR France [TIAB] OR Georgia [TIAB] OR Germany [TIAB] OR Greece [TIAB] OR Hungary [TIAB] OR Iceland [TIAB] OR Ireland [TIAB] OR Israel [TIAB] OR Italy [TIAB] OR Kazakhstan [TIAB] OR Kosovo [TIAB] OR Kyrgyzstan [TIAB] OR Latvia [TIAB] OR Lithuania [TIAB] OR Luxembourg [TIAB] OR Malta [TIAB] OR Monaco [TIAB] OR Montenegro [TIAB] OR Netherlands [TIAB] OR Macedonia [TIAB] OR Norway [TIAB] OR Poland [TIAB] OR Portugal [TIAB] OR Moldova [TIAB] OR Romania [TIAB] OR Russia [TIAB] OR "San Marino" [TIAB] OR Serbia [TIAB] OR Slovakia [TIAB] OR Slovenia [TIAB] OR Spain [TIAB] OR Sweden [TIAB] OR Switzerland [TIAB] OR Tajikistan [TIAB] OR Turkey [TIAB] OR Turkmenistan [TIAB] OR Ukraine [TIAB] OR "United Kingdom" [TIAB] OR Britain [TIAB] OR Ireland [TIAB] OR Uzbekistan [TIAB] | | | 979,678 | 4:55:42 |
| 14 | ("Europe"[Mesh]) OR "Europe, Eastern"[Mesh] | | | 1,489,073 | 4:52:08 |
| 13 | policy maker [TIAB] OR policy makers [TIAB] policy making [TIAB] OR evidence-based practice [TIAB] OR evidence based program [TIAB] OR evidence based programs [TIAB] OR evidence informed practice [TIAB] OR evidence informed program [TIAB] OR evidence informed programs [TIAB] | | | 14,244 | 4:50:11 |
| 11 | "Public Policy"[Mesh:NoExp] OR "Health Policy"[Mesh:NoExp] OR "Policy Making"[Mesh:NoExp] OR "Administrative Personnel"[Mesh:NoExp] | | | 123,804 | 4:46:10 |
| 10 | #1 OR #2 OR #3 OR #4 OR #5 OR #6 OR #7 OR #8 | | | 304,404 | 4:45:31 |
| 8 | evidence brief [TIAB] OR evidence briefs [TIAB] OR evidence summary [TIAB] OR evidence summaries [TIAB] OR policy brief [TIAB] OR policy briefs [TIAB] OR deliberative dialogue [TIAB] OR deliberative dialogues [TIAB] OR policy dialogue [TIAB] OR policy dialogues [TIAB] OR registry [TIAB] OR registries [TIAB] OR repositories [TIAB] OR repository [TIAB] OR portals [TIAB] OR e-libraries [TIAB] OR discussion forum [TIAB] OR discussion forums [TIAB] OR electronic libraries [TIAB] OR blog* [TIAB] OR microblog* [TIAB] OR weblog* [TIAB] | | | 172,094 | 4:44:41 |
| 7 | reporting system [TIAB] OR reporting systems [TIAB] OR tracking system [TIAB] OR tracking systems [TIAB] | | | 15,045 | 4:43:16 |
| 6 | evidence management [TIAB] OR evidence creation [TIAB] OR evidence storage [TIAB] OR evidence storing [TIAB] OR evidence exchange [TIAB] OR evidence exchanging [TIAB] OR evidence sharing [TIAB] OR evidence transfer [TIAB] OR evidence transferring [TIAB] OR evidence translation [TIAB] OR evidence generation [TIAB] OR evidence generating [TIAB] OR evidence mobilization [TIAB] OR evidence mobilisation [TIAB] OR evidence integration [TIAB] OR evidence application [TIAB] OR evidence brokering [TIAB] OR evidence dissemination [TIAB] OR disseminating evidence [TIAB] OR disseminating knowledge [TIAB] OR evidence acquisition [TIAB] | | | 27,119 | 4:19:08 |
| 5 | communities of practice [TIAB] OR community of practice [TIAB] | | | 1,401 | 4:16:59 |
| 4 | knowledge network* [TIAB] OR knowledge platform [TIAB] OR knowledge platforms [TIAB] OR knowledge repository [TIAB] OR knowledge database [TIAB] OR research network* [TIAB] OR research platform [TIAB] OR research platforms [TIAB] OR research repository [TIAB] OR research database [TIAB] OR information network* [TIAB] OR information platform [TIAB] OR information platforms [TIAB] OR information repository [TIAB] OR information database [TIAB] | | | 20,563 | 4:16:24 |
| 3 | data management [TIAB] OR data creation [TIAB] OR data storage [TIAB] OR data storing [TIAB] OR data exchange [TIAB] OR data exchanging [TIAB] OR data sharing [TIAB] OR data transfer [TIAB] OR data transferring [TIAB] OR data translation [TIAB] OR data generation [TIAB] OR data generating [TIAB] OR data mobilization [TIAB] OR data mobilisation [TIAB] OR data integration [TIAB] OR data application [TIAB] OR data brokering [TIAB] OR data dissemination [TIAB] OR data acquisition [TIAB] | | | 37,293 | 4:15:57 |
| 2 | information management [TIAB] OR information creation [TIAB] OR information storage [TIAB] OR information storing [TIAB] OR information exchange [TIAB] OR information exchanging [TIAB] OR information sharing [TIAB] OR information transfer [TIAB] OR information transferring [TIAB] OR information translation [TIAB] OR information generation [TIAB] OR information generating [TIAB] OR information mobilization [TIAB] OR information mobilisation [TIAB] OR information integration [TIAB] OR information application [TIAB] OR information brokering [TIAB] OR information dissemination [TIAB] OR information acquisition [TIAB] | | | 23,061 | 4:15:48 |
| 1 | "Knowledge Management"[Mesh] OR "Translational Medical Research"[Mesh] OR "Social Networking"[Mesh:NoExp] | | | 16,748 | 4:14:12 |

## EMBASE search strategy

No. Query Results

#21. #13 AND #18 AND #19 AND [2005-2021]/py

#20. #13 AND #18 AND #19

#19. europe*:ti,ab OR albania:ti,ab OR andorra:ti,ab

OR armenia:ti,ab OR austria:ti,ab OR

azerbaijan:ti,ab OR belarus:ti,ab OR

belgium:ti,ab OR 'bosnia and herzegovina':ti,ab

OR bulgaria:ti,ab OR croatia:ti,ab OR

cyprus:ti,ab OR czechia:ti,ab OR denmark:ti,ab OR

estonia:ti,ab OR finland:ti,ab OR france:ti,ab OR

georgia:ti,ab OR germany:ti,ab OR greece:ti,ab OR

hungary:ti,ab OR iceland:ti,ab OR israel:ti,ab OR

italy:ti,ab OR kazakhstan:ti,ab OR kosovo:ti,ab

OR kyrgyzstan:ti,ab OR latvia:ti,ab OR

lithuania:ti,ab OR luxembourg:ti,ab OR

malta:ti,ab OR monaco:ti,ab OR montenegro:ti,ab

OR netherlands:ti,ab OR macedonia:ti,ab OR

norway:ti,ab OR poland:ti,ab OR portugal:ti,ab OR

moldova:ti,ab OR romania:ti,ab OR russia?:ti,ab

OR 'san marino':ti,ab OR serbia:ti,ab OR

slovakia:ti,ab OR slovenia:ti,ab OR spain:ti,ab

OR sweden:ti,ab OR switzerland:ti,ab OR

tajikistan:ti,ab OR turkey:ti,ab OR

turkmenistan:ti,ab OR ukraine:ti,ab OR 'united

kingdom':ti,ab OR britain:ti,ab OR ireland:ti,ab

OR uzbekistan:ti,ab

#18. #14 OR #15 OR #16 OR #17

#17. (evidence NEXT/1 (based OR informed) NEXT/1

(practice OR program? OR decision OR

decisions)):ti,ab

#16. policy:ti,ab OR policies:ti,ab

#15. policymaking:ti,ab OR 'decision making':ti,ab

#14. 'public policy'/de OR 'health care policy'/de OR

'policy maker'/de

#13. #1 OR #2 OR #3 OR #4 OR #5 OR #6 OR #7 OR #8 OR

#9 OR #10 OR #11 OR #12

#12. 'online social network'/de

#11. 'knowledge management'/de

#10. 'knowledge translation'/exp

#9. (electronic NEXT/1 libraries):ti,ab,kw

#8. (discussion NEXT/1 forum$):ti,ab,kw

#7. registries:ab,ti OR repositories:ab,ti OR

repository:ab,ti OR portals:ab,ti OR 'e

libraries':ab,ti

#6. ((deliberative OR policy) NEXT/2

dialogue$):ti,ab,kw

#5. ((evidence OR policy) NEXT/2 brief$):ti,ab,kw

#4. ((reporting OR tracking) NEXT/2 system$):ti,ab,kw

#3. (communit* NEXT/2 practice):ti,ab,kw

#2. ((knowledge OR research OR information) NEXT/2

(network* OR platform OR repository OR

database)):ti,ab,kw

#1. ((knowledge OR information OR data OR evidence)

NEAR/2 (management OR creation OR storage OR

storing OR exchang* OR sharing OR transfer* OR

translation OR generation OR generating OR

mobili$ation OR integration OR application OR

brokering OR dissemination OR

acquisition)):ti,ab,kw

.......................................................

## CENTRAL

ID Search Hits

#1 (((knowledge OR information OR data OR evidence) NEAR/2 (management OR creation OR storage OR storing OR exchang* OR sharing OR transfer* OR translation OR generation OR generating OR mobili$ation OR integration OR application OR brokering OR dissemination OR acquisition))):ti,ab,kw (Word variations have been searched) 11929

#2 (((knowledge OR research OR information) NEXT/2 (network* OR platform OR repository OR database))):ti,ab,kw (Word variations have been searched) 2440

#3 (communit* NEXT/2 practice):ti,ab,kw (Word variations have been searched) 617

#4 ((reporting OR tracking) NEXT/2 system$):ti,ab,kw 620

#5 ((evidence OR policy) NEXT/2 brief$):ti,ab,kw 103

#6 ((deliberative OR policy) NEXT/2 dialogue$):ti,ab,kw 5

#7 (registries OR repositories OR repository OR portals OR 'e libraries'):ti,ab,kw 3964

#8 (discussion NEXT/1 forum$):ti,ab,kw 119

#9 (electronic NEXT/1 libraries):ti,ab,kw 0

#10 MeSH descriptor: [Translational Medical Research] this term only 124

#11 MeSH descriptor: [Social Networking] explode all trees 121

#12 (blog* or microblog* or weblog*):ti,ab,kw 176

#13 MeSH descriptor: [Policy] explode all trees 831

#14 MeSH descriptor: [Policy Making] explode all trees 51

#15 MeSH descriptor: [Health Policy] explode all trees 672

#16 (policy* or policies or decision*):ti,ab,kw 43024

#17 (evidence NEXT/1 (based OR informed) NEXT/1 (practice OR program$)):ti,ab,kw 3121

#18 #1 OR #2 OR #3 OR #4 OR #5 OR #6 OR #7 OR #8 OR #9 OR #10 OR #11 OR #12 19675

#19 #13 OR #14 OR #15 OR #16 OR #17 45830

#20 #18 AND #19 2898

#21 (Europe* OR Albania OR Andorra OR Armenia OR Austria OR Azerbaijan OR Belarus OR Belgium OR "Bosnia and Herzegovina" OR Bulgaria OR Croatia OR Cyprus OR Czech* OR Denmark OR Estonia OR Finland OR France OR Georgia OR Germany OR Greece OR Hungary OR Iceland OR Ireland OR Israel OR Italy OR Kazakhstan OR Kosovo OR Kyrgyzstan OR Latvia OR Lithuania OR Luxembourg OR Malta OR Monaco OR Montenegro OR Netherlands OR Macedonia OR Norway OR Poland OR Portugal OR Moldova OR Romania OR Russia? OR "San Marino" OR Serbia OR Slovakia OR Slovenia OR Spain OR Sweden OR Switzerland OR Tajikistan OR Turkey OR Turkmenistan OR Ukraine OR "United Kingdom" OR Britain or Ireland OR Uzbekistan):ti,ab,kw (Word variations have been searched) 122013

#22 #20 AND #21 438

## OpenGrey

Access through: <https://easy.dans.knaw.nl/ui/datasets/id/easy-dataset:200362>

(“knowledge management” OR “knowledge creation” OR “knowledge storage” OR “knowledge storing” OR “knowledge exchange” OR “knowledge exchanging” OR “knowledge sharing” OR “knowledge transfer” OR “knowledge transferring” OR ‘knowledge translation” OR “knowledge generation” OR “knowledge generating” OR “knowledge mobilization” OR “knowledge mobilization” OR “knowledge integration” OR “knowledge application” OR “knowledge brokering” OR “knowledge dissemination”)

- Filtered by “Health Sciences” and “Political and administrative sciences”
