# Supplementary material for: Knowledge management tools and mechanisms for evidence-informed decision-making in the WHO European Region: a scoping review
Source: Health Res Policy Syst. 2023 Oct 31;21:113. doi: 10.1186/s12961-023-01058-7 (PMC10619313; doi:10.1186/s12961-023-01058-7)
Supplement: Supplementary file 3 — Additional file 3: Appendix 3. Table of characteristics - Indicators. [file 12961_2023_1058_MOESM3_ESM.docx]

**Studies on Indicators (n=9)**

| **Author, Year** | **Country** | **Study design** | **KM tool/Program** | **Policy Outcome(s)** | **Main Results**  **Is the intervention effective overall? (yes/no/inconclusive)** | **Implementation considerations** |
| --- | --- | --- | --- | --- | --- | --- |
| Costa 2019 | Regional | Retrospective analysis | EURO-HEALTHY, a Population Health Index (PHI), (39 indicators)  Knowledge storage | Agenda-setting | Inform regional policies by providing evidence on relevant dimensions where policy action has high potential for reducing inequalities in health between regions. | **Availability of data**  *“Although the missing data on the set of the PHI indicators was significant, the mean availability score for the EURO-HEALTHY PHI indicators is 0.8 and the regional availability score is 0.7, which reveal the strength of the indicators as well as the data completeness protocol for missing data”.* |
| Bogaert 2018 | Regional | Survey | Healthy Life Years (HLY) - a disability-free life expectancy  the Global Activity Limitation Indicator (GALI) | Health strategies and plans | HLY are often used to set targets and develop strategies in health such as national health plans and design policies and programs.  Analysis of the HLY have:   - led to policy changes in e.g. Estonia. - discussed in high level political meetings in countries such as France and Italy, and at the European Commission, in the context of the European Pillar of Social Rights where HLY are a headline indicator. - In Lithuania, HLY have even taken over as the main evaluation criteria in the program of the government. - the GALI and HLY have been used for policy targets outside the health sector such as in the area of pension and retirement age or in the context of sustainable development. | **Increase the capacity** to analyse HLY within the EU through training workshops and guidelines for estimation and interpretation of HLY.  GALI question is institutionalized in most important health questionnaires through the MEHM such as the EU-SILC, the SHARE and the EHIS |
| Tudisca 2018 | six European countries (Denmark, Finland, Italy, the  Netherlands, Romania, the United Kingdom) | Delphi technique | measurable indicators for evidence-informed policy-making developed by ‘Research into Policy to enhance Physical Activity’ (REPOPA) project | Evidence-informed policymaking | 25 indicators were validated, covering EIPM issues related to human resources, documentation, participation and monitoring, and stressing different levels of  knowledge exchange and involvement of researchers and other stakeholders in policy development and evaluation | Institutionalization of the indicators  existing evidence and tools for EIPM should be available in local languages and sharing lessons and learning from country experiences is important as an action to build EIPM capacities |
| Verschuuren, 2013 | Regional | observational | ECHIM (European Community Health Indicators & Monitoring) | Policy and planning | High quality health information (informed by ECHI) serves the EU and Member States by helping to direct health, welfare and other policies and planning toward meeting peoples’ health needs. | Standards for data collection, reporting mechanisms  Sustained efforts at EU and national level are needed to keep the system functional and up to date. |
| Fehr 2018 | Regional | survey | European Core Health Indicators (ECHI) | Policy evaluation and prioritization | ECHI-indicator knowledge and expertise and strengthening the scientific base that supports the effective development and use of health indicators for health policy evaluation and prioritization by the EU and its Member States.  The ECHI shortlist can contribute to the collection of comparable policy-relevant health data in Europe, foster evidence-based public health and contribute to Member States learning from each other. | ***Policy relevance*** is an essential but not systematically developed criterion for the inclusion of indicators into the ECHI shortlist.  **Data availability** *is crucial for the actual implementation of indicators and has considerably increased for ECHI in the last decade.* |
| Berler 2005 | Greece | Case study | Key Performance Indicators | Health systems performance | *“The proposed KPIs are forming a complete set of metrics that enable the performance management of a regional health-care system.”* |  |
| Devleesschauwer 2014 | Belgium | Opinion piece | Disability-Adjusted Life Year (DALY) | Evidence-based public health policy | DALYs are a highly valuable measure to set priorities for public health research and policy by quantifying the total disease burden and the contribution of different diseases and risk factors | Most DALY estimations remained academic exercises, with little or no direct knowledge transfer to the concerned policy instances.  Global estimates might not be grounded with the best available local data, with no ownership and timelines of these global estimates |
| Colzani 2017 | Sweden | Modelling study | Disability-Adjusted Life Year (DALY) | The burden of disease framework enables the assessment of the health impact of diseases | DALYs provide a summary of the impact of communicable diseases, forming the burden of disease framework toolkit |  |
| Kokko, 2021 | Finland | Qualitative study | National Health Indicators (NHIs) | NHIS allow the assessment of the national healthcare system | NHIs allow to measure outcomes in order to improve policy making in healthcare |  |
