# Supplementary material for: Knowledge management tools and mechanisms for evidence-informed decision-making in the WHO European Region: a scoping review
Source: Health Res Policy Syst. 2023 Oct 31;21:113. doi: 10.1186/s12961-023-01058-7 (PMC10619313; doi:10.1186/s12961-023-01058-7)
Supplement: Supplementary file 4 — Additional file 4: Appendix 4. Table of characteristics - Surveys. [file 12961_2023_1058_MOESM4_ESM.docx]

**Surveys (n=3)**

| **Author, Year** | **Country** | **Study design** | **KM tool/Program** | **Policy Outcome(s)** | **Main Results**  **Is the intervention effective overall? (yes/no/inconclusive)** | **Implementation considerations** |
| --- | --- | --- | --- | --- | --- | --- |
| Cavill 2006 | Europe | Case Study | Survey | Policy development and promotion | Surveys and the assessment of the scale of the problem allows calculation of the population attributable risk, and identification of groups with higher levels of risk, influencing policy development for promoting physical activity | **Systematic data collection** |
| Tolonen 2018 | Europe | Observational | European health examination surveys (HES) | Evidence-informed policymaking, planning and evaluation of prevention programs | The European health examination surveys (HES) have the potential to identify priorities health problems and can be used for health monitoring. | Ensuring **standardization** of the data collection is essential to allow HES data to be comparable across EU countries and over time |
| Volen 2021 | Bulgaria | Case Study | Randomized control trial | National policy development | A randomized control trial, as part of the Springboard for School Readiness (SSR) project assessing the impacts of kindergarten participation on child development, later used as evidence to push for the removal of attendance fees, improving participation rate and positively influencing child development. | -- |
