# Supplementary material for: Knowledge management tools and mechanisms for evidence-informed decision-making in the WHO European Region: a scoping review
Source: Health Res Policy Syst. 2023 Oct 31;21:113. doi: 10.1186/s12961-023-01058-7 (PMC10619313; doi:10.1186/s12961-023-01058-7)
Supplement: Supplementary file 5 — Additional file 5: Appendix 5. Table of characteristics - Registries. [file 12961_2023_1058_MOESM5_ESM.docx]

**Studies on registries (n=21)**

| **Author, Year** | **Country** | **Study design** | **KM tool/Program** | **Policy Outcome(s)** | **Main Results**  **Is the intervention effective overall? (yes/no/inconclusive)** | **Implementation considerations** |
| --- | --- | --- | --- | --- | --- | --- |
| UK Renal Registry, 2009 | UK | Annual report | UK Renal Registry  Knowledge storage | Resource allocation and health service planning  Policy formulation | The UKRR provides a cost-effective source of detailed information on renal services and allow a prediction of the need for renal replacement therapy facilities | -- |
| Ludvigsson 2016 | Sweden | Case study | The Population Register  The Total Population Register (TPR)  Knowledge storage | Government decisions (planning and resource allocation)  Policy implementation | Can be used as the basis for correct decisions and measures by government and other regulatory authorities such as taxation, allowance, and subsidies. | -Quality and timeliness of the Swedish population registers  -Confidentiality |
| Wojcieszak 2014 | Poland | Retrospective analysis | The Polish National Cancer Registry (KRN)  Knowledge storage | establishment of public health priorities and health care planning (Resource allocation)  Agenda-setting | The study revealed that uro-oncological reporting in Poland is not perfect and that the KRN is not a fully credible source of data on the incidence of renal cancer. | Completeness and accuracy  Computerization makes it possible to capture all cancer cases in the registry. |
| Rossi 2016 | Regional | Case study | European Register of Cystic Echinococcosis (ERCE)  Knowledge storage | Acknowledge problem’s magnitude  Agenda-setting  Support implementation of policies  Policy implementation | Data collected in ERCE will allow national authorities and international agencies such as the European Centre for Disease Prevention and Control (ECDC) to acknowledge the magnitude of the problem by reporting cases otherwise not captured by systems such as hospital discharge records.  Support with data for planning and implementation of public health policies toward disease management and control. | Lack of mandatory notification requirement  re-evaluation of the case definition for ‘echinococcosis’  better designed reporting systems  harmonize data collection, monitoring and reporting of CE, according to EU legislation |
| Sharp, 2014 | Ireland | Retrospective cohort | National Cancer Registry  Knowledge storage | Planning of services | Registries can provide a population-based estimate of the number of cancer survivors, information likely to be of considerable value to service planners and providers in the statutory and voluntary sectors. They also reveal important heterogeneity within the survivor population which is likely to determine their ongoing medical and supportive care needs and hence influence service requirements and provide an indication of the likely magnitude of groups of survivors who may have specific service and support needs. | -- |
| Siesling 2015 | Multiple European countries | Survey | Cancer registries  Knowledge storage | Improvement of cancer care | Improvement of cancer care projects was equally supported by only 8% of cancer registries, but ad hoc by 62%.  Evaluation of mass screening for cancer was regularly supported by 44% and ad hoc by 42% of the responding cancer registries. | -- |
| Murillo 2016 | Ireland | Descriptive | Registry for autism patients  Knowledge storage | inform policy and programs | inform policy and programs aimed at improving the public health of Ireland’s autism community. | -- |
| Ornerheim 2018 | Sweden | Opinion piece | National quality registries  Knowledge storage | Policy formulation | Self-reinforcing actions have strengthened the NQR development and are of importance in the survival of the NQR idea. This development, together with technical progress, opened a window of opportunity that gave rise to the change in policy towards transparency and RCs as an unintended consequence. | -- |
| UK Renal Registry, 2017 | United Kingdom | Technical report | UK renal registry  Knowledge storage | Quality assessment  Prediction of the need of services | Data may identify a potential unmet need in the population and permit assessment on the equity of service provision. In the future, the UKRR database should also provide information on nephrology and pre-dialysis patients and allow a prediction of the need for renal replacement therapy facilities. | -- |
| Mohseninejad 2015 | Netherlands | Case study | Colon cancer patients’ registries  Knowledge storage | Reimbursement | Use of registry data to support the access with evidence process for the reimbursement of oxaliplatin for stage III colon cancer treatment | -- |
| Mohammadzadeh 2020 | Multiple European countries | Comparative/Descriptive | MS registries  Knowledge storage | Care planning | Knowledge of the incidence, survival, and  death rate of MS disease enables health authorities and policymakers to identify  at-risk groups and provide regular care for them. | -- |
| Stanimirovic 2019 | Multiple European countries | Case study | Rare disease registry  Knowledge storage | Management of rare diseases | Quality rare disease registries provide a beneficial and applicable platform in all stages of evidence-informed healthcare policymaking, and may contribute to significant advancement in the management of rare diseases. | -- |
| Mandavia 2018 | United Kingdom | Qualitative study | national registry of auditory implants  Knowledge storage | Regulation, decision-making, and guidelines | Strengthening the evidence base and regulation of auditory implants, driving quality and safety improvements, increased transparency, facilitating patient decision-making and informing policy and guidelines development. | -- |
| Taruscio 2014 | Italy | Descriptive | The Italian National Rare Diseases Registry  Knowledge storage | policy making and health services planning | The main objective is producing epidemiologic evidence on rare diseases and supporting policy making and health services planning. | Data quality still represents a limitation for any sound epidemiological estimate of rare diseases in Italy |
| Stanimirovic, 2020 | Slovenia | Case study | Rare disease registry  Knowledge storage | Evidence-informed policymaking | Evidence-informed policymaking | Methodology for the development of a registry for Rare Diseases  Recommendations of the focus group participants and steps from the literature: -The registry’s purpose  -Key stakeholders and the feasibility of the registry  -Registry team -Registry scope and data set  -Technological implementation |
| Jonker 2021 | Multiple European countries | Survey | Rare Disease Registries  Knowledge storage | support regulatory decision making | -- | Stakeholders including employees working in the pharmaceutical industry, regulators, academia, registry owners, and patient representatives considered that 30% of source data must be verified and 20% of missing data would provide acceptable levels of data quality |
| McGettigan 2019 | United Kingdom | Qualitative study | Patient Registries  Knowledge storage | Support regulatory decision-making | -- | Registry funding and support may be limited, causing difficulties in maintaining database quality assurance processes and trainings.  Linkages to external databases such as prescription dispensing, employment, or death registries, when possible, may add to the value of registry data. |
| Pricci 2019 | Italy | Opinion pieces/editorials/commentaries | Computerized National Registry of Growth Hormone therapy | Policy making | The interface allows obtaining and managing correct and complete data to provide public health surveillance on GH therapy, both at national and local levels, necessary for policymakers decisions. | -- |
| Carinci 2015 | Italy | Case study | Integrated diabetes registries | Information sharing | *“Improve quality of care through continuous monitoring of outcomes and prompt identification of subjects at increased risk of developing complications”* |  |
| Allen 2022 | Regional | case study | “Registry Evaluation and Quality Standards Tool” (REQueST). | Decision making | REQueST tool aims at evaluating clinical registries to maximize their utility by HTA bodies | -- |
| Capozzi 2018 | Italy | Retrospective analysis | AIFA Registry | Drugs reimbursement | -- | *“the reimbursement process*  *of biologics in gastrointestinal oncology can be improved*  *when a health policy reimbursement professional Pharmacist is integrated in the multidisciplinary team along with clinicians.”* |
