# Supplementary material for: Knowledge management tools and mechanisms for evidence-informed decision-making in the WHO European Region: a scoping review
Source: Health Res Policy Syst. 2023 Oct 31;21:113. doi: 10.1186/s12961-023-01058-7 (PMC10619313; doi:10.1186/s12961-023-01058-7)
Supplement: Supplementary file 6 — Additional file 6: Appendix 6. Table of characteristics - Surveillance and Observatories. [file 12961_2023_1058_MOESM6_ESM.docx]

**Surveillance and Observatory (n=24)**

| **Author, Year** | **Country** | **Study design** | **KM tool/Program** | **Policy Outcome(s)** | **Main Results**  **Is the intervention effective overall? (yes/no/inconclusive)** | **Implementation considerations** |
| --- | --- | --- | --- | --- | --- | --- |
| Schonning 2021 | Denmark | Narrative/literature review | Danish Microbiology Database used for surveillance | Response to COVID-19 | Support the national policy toward the COVID-19 epidemic. | -- |
| Paternoster 2017 | Italy | Estimation study | West Nile virus surveillance | Support policy | Guide public health interventions to mitigate the risk of West Nile virus transmission via blood transfusion | -- |
| Grabenhenrich, 2021 | Germany | Descriptive study | Real-time surveillance | Evaluation of health community interventions | The data enable continuous monitoring of the spatio-temporal distribution of relevant health phenomena and serve to analyze community interventions or population-wide measures. | -- |
| Sartor 2020 | Italy | Case study | Surveillance systems | Public health policies | Analyze the pandemic evaluation and influence public health policies | -- |
| Lavis 2013 | England, Norway, and Spain | Book | European Observatory on Health Systems and Policies | Framing of policies | The European Observatory on Health Systems and Policies adopted in England, Norway, and Spain provides faming policy options for the future funding of long-term care, through packaging and sharing information with policymakers. It also supports the development of several health-related policies such as anti-tobacco policies.  Its direct role includes supporting the work of a national coalition for smoking prevention, identifying and supporting members who could bring their expertise to bear on the issues at hand, and mediating discussions among different groups. | -- |
| Stroetmann, 2011 | European Union and USA | Policy brief | Observatory | -- | Develop and promote common methods for responding to global eHealth challenges | -- |
| Busse 2018 | Multinational | Opinion pieces/editorials/commentaries | European Observatory | -- | Observatory has four core functions: country monitoring, analysis, comparative health systems performance assessment, and knowledge brokering. | -- |
| Egan 2007 | European region | Literature review | Food composition data in Europe | Nutritional policy | Support nutritional policy making in the European Union | **Data harmonization** is not solely a technical issue, but also involves creating durable and sustainable structures to maintain the viability of the data. |
| Diou 2021 | Athens, Larissa and Thessaloniki in Greece, the Stockholm area in Sweden and Dublin in Ireland. | Case study | BigO | Policy formulation | Support public health authorities in formulating effective, context-specific policies and interventions addressing childhood obesity. | Acceptability of the interventions |
| Gutenberg 2018 | UK | Case study | Big Data | support public health policy making | Provide a platform  to support public health policy making for hearing impairment. | -- |
| Heitmueller 2014 | UK | Commentary | Big Data | Public health policy making | provides a platform to support public health policy making | Making the case for data sharing requires evidence that changes deliver the promised benefits. Other facilitators for uptake include leading by example, creating demand and capability, devolving responsibility, creating trust networks, and legislating smartly |
| Fleming 2014 | UK | Descriptive | Data mashup | Decision support | Provide both the evidence base and the decision support tools | *Potential challenges with the use of data mashups include: access to and ownership of original data, training of personnel and users, rapidly changing hardware and software, funding and resources, confidentiality and standardization of data, and interactions with variety of stakeholders*. |
| Ziese 2017 | Germany | Case Study | Health surveillance system | Public health policy making | Health surveillance system provides consistent answers to health policy issues | -- |
| Lauriola 2020 | Italy | Narrative/literature review | Environmental and Public Health Tracking (EPHT) | Partaking recent global EPHT activities and resources | EPHT aims to merge and interpret environmental hazards and health data to provide information for public health policy-makers to reduce the environmental burden of disease. | -- |
| O'Reilly 2020 | United Kingdom | Case Study | The Administrative Data Research Northern Ireland (ADR NI) Database | Improve the access to and use of administrative data for legislation | ADRNI uses research based on administrative data to influence policy making | -- |
| Sultana 2020 | Italy | Narrative/literature review | Healthcare databases | Answer public health questions of decision-makers through the availability of evidence | Data sources can provide regulatory agencies with evidence that can inform the development of regulatory interventions and policies | -- |
| Vardas 2016 | Regional (EU) | Journal Article | Atlas of Cardiology | Collecting data to inform policy making | Atlas of Cardiology aims to develop an extensive data for promoting evidence-based health policy making in European cardiology and thus reduce the burden of disease | -- |
| Cavelaars 2010 | Regional (EU) | Case Study | Nutri-RecQuest | Evidence-based recommendations | Nutri-RecQuest provides access to efficient search functions that are valuable for decision-makers for developing recommendations on nutrient requirements | -- |
| Romero-Lopez-Alberca 2019 | Spain | Meta analysis | ESMS/DESDE (The European Service Mapping Schedule/ Description and Evaluation of Services and DirectoriEs) | Evidence-informed policy | The ESMS/DESDE system allows the assessment of health and social systems and the comparison of data across and within countries, influencing policy-making | ESMS/DESDE requires extensive training and the interpretation of the results by decision makers should involve additional support from experts. |
| Couch 2013 | UK | Narrative/literature review | e-lab Stock of Health | Policy Decision Support | The Stock of Health harnesses emerging data to be used for the dissemination of health policy models that support policy making in Cardiovascular disease (CVD) | -- |
| Seabra, 2021 | Portugal | Case study | The Portuguese Tuberculosis Surveillance System – SVIG-TB | Public Policies | The Portuguese Tuberculosis Surveillance System – SVIG-TB have been important in guiding policies for the elimination of diseases within the national Tuberculosis program | -- |
| Achterberg 2008 | Regional | Case study | EUPHIX website lies a complex internet application, including databases, i.e. for indicator data, for internal and external web links and for references and definitions. | Policy-making | The system aimed at providing policy-relevant data, information and knowledge on Public Health across the EU and was designed to be integrated in the Health-EU Portal. | A web-based information system, however, only makes sense if it is maintained and updated in a sustainable way  Financial sustainability |
| Blessings 2017 | Regional | Report | The WHO European Health Information Gateway | Policy-making | Provide access in a format that policy-makers can easily understand. | -- |
