# Supplementary material for: Knowledge management tools and mechanisms for evidence-informed decision-making in the WHO European Region: a scoping review
Source: Health Res Policy Syst. 2023 Oct 31;21:113. doi: 10.1186/s12961-023-01058-7 (PMC10619313; doi:10.1186/s12961-023-01058-7)
Supplement: Supplementary file 7 — Additional file 7: Appendix 7. Table of characteristics - HIS. [file 12961_2023_1058_MOESM7_ESM.docx]

**Studies on Health information Systems (n=4)**

| **Author, Year** | **Country** | **Study design** | **KM tool/Program** | **Policy Outcome(s)** | **Main Results**  **Is the intervention effective overall? (yes/no/inconclusive)** | **Implementation considerations** |
| --- | --- | --- | --- | --- | --- | --- |
| Bogaert 2018 | regional | Case study | European Research Infrastructure Consortium on Health Information  for Research and Evidence-based Policy (HIREP-ERIC) | Policy making | An infrastructure that facilitates interaction of networks and experts in health information and should provide easy access to high quality and comparable data for purposes of research and policy making, and focus its activities around generating, managing, exchanging and translating health information. | A well-defined and sustainable EU health information system infrastructure as opposed to diversity and fragmentation  Budgetary restrictions High pressure workload Health information gaps Competing initiatives |
| Bogaert 2017 | regional | A stakeholder consultation | current EU health information system | evidence-based policy-making | No single, integrated and sustainable EU-wide public health monitoring system or health information system exists  BRIDGE Health is working towards an EU health information and data generation network covering major EU health policy areas. | A better system is about sustainability, better coordination, governance and collaboration  among national health information systems and stakeholders to jointly improve, harmonize, standardize and analyze  health information. |
| Bozorgmehr 2019 | Regional | Policy brief | Health information system | Integrating refugees and migrant health data into health information systems | Data on refugee and migrant health were available in 25 of the 53 Member States of the WHO European Region, but the extent of availability, type of data and main data sources differed greatly.  Data sources that recorded data on refugee and migrant health were diverse and their integration into national health information systems was limited, except for population registers, notification systems and medical records in some countries. | Such data are not always systematically collected by country health information systems in the WHO European Region. |
| Pérez Sust, 2020 | Spain | Case study | Information Communication and Technology (ICT) | Strengthen health system | *“capacity of digital health technologies to increase the efficiency of health care systems”* | ICT-enabled solutions should be supplemented by financial incentives for health providers |
