# Supplementary material for: Knowledge management tools and mechanisms for evidence-informed decision-making in the WHO European Region: a scoping review
Source: Health Res Policy Syst. 2023 Oct 31;21:113. doi: 10.1186/s12961-023-01058-7 (PMC10619313; doi:10.1186/s12961-023-01058-7)
Supplement: Supplementary file 8 — Additional file 8: Appendix 8. Table of characteristics - Evidence Synthesis. [file 12961_2023_1058_MOESM8_ESM.docx]

**Studies on Evidence Synthesis (n=19)**

| **Author, Year** | **Country** | **Study design** | **KM tool/Program** | **Policy Outcome(s)** | **Main Results**  **Is the intervention effective overall? (yes/no/inconclusive)** | **Implementation considerations** |
| --- | --- | --- | --- | --- | --- | --- |
| Wilson 2017 | UK | CBA | Demand-led evidence briefing service | Use of research evidence by commissioners  Policy formulation | Evidence briefing service was not associated with increases in NHS commissioners capacity to acquire, assess, adapt and apply research evidence to support decision-making, individual intentions to use research findings or perceptions of commissioners relationships with researchers. | Resource-intensive |
| Eriksson 2017 | Sweden | Narrative review | An early awareness and alert (EAA) system  (HTA tool) | Resource allocation  Policy implementation | the outputs of the Swedish EAA System are important for the prioritization of how resources  are used.  the EAA System was incorporated into the Swedish national process for managed introduction and follow-up of new medicines. | -- |
| Anderson 2020 | England | Systematic review | Scoping literature reviews | Redesign Children and Adolescent Mental Health Services  Policy formulation and implementation | The development of the Local Transformation Plan that guided the subsequent local CAMHS redesign of East of England Children and Adolescent Mental Health Services (CAMHS) | Enhance methodological robustness, minimize bias, and ensure reliability of findings. |
| Parmelli 2013 | Regional | Case study | DECIDE  (Developing and Evaluating Communication strategies to support Informed Decisions and practice based on Evidence) | Coverage decisions  Policy adoption and formulation | Introduce or discontinue the financial coverage of a certain drug, test, device, service or intervention program. | -- |
| Seidler 2021 | Regional  (Germany, Switzerland, Austria) | Case study | Rapid reviews | COVID-19 policies | Rapid reviews can form an important basis for evidence-based policy advice for COVID-19 | A standardized approach (automation, one reviewer, use of GRADE methodology, increased search specificity |
| Jönsson 2013 | Regional | Case study | HTA | Resource allocation | Inform decisions about allocation of resources between competing demands. The aim is to create the most value for money, directing spending on technologies that give the most benefit for the populations that the systems serve, which also pay for the services through taxes. | -- |
| Fredriksson 2014 | Sweden | Qualitative document analysis | Evidence-based National Guidelines | Resource allocation  Priority setting | Support for prioritizations and decision-making on how to allocate resources within healthcare according to population need and decisions on local and regional healthcare programs. | -- |
| Kornør 2015 | Norway | Case study | Dissemination of plain language summaries of systematic reviews | Child welfare services | Inform child welfare services by the high-quality evidence | Ensure the contextualization of evidence and the plain-language format summaries |
| Busert 2018 | Germany, Austria and Switzerland | User testing through interviews | German language summary format for systematic reviews | Decision-making | support the knowledge transfer to public health decision-makers | participants particularly appreciated receiving information in their own language and the key messages and the relevance for public health practice.  several barriers include: information-dense structure and difficulties with understanding statistical terms, lack of actionable recommendations and contextual information. |
| Stansfield 2018 | England | Case study | Knowledge translation framework | support evidence into practice | Supports the synthesis of knowledge into national policy and local practice and focuses on partnership between the researcher and the decision-maker in providing up to date evidence in a relevant and appropriate format to inform policy and practice | Involving communities in translating evidence into practice  Producing evidence synthesis should be coupled by KT activities for dissemination and communication |
| van der Graaf 2018 | United Kingdom | Qualitative | Localized and tailored research evidence | Shape decision-making process | Localizing published research evidence and tailoring it into actionable messages are the two pillars of knowledge mobilizations | -- |
| Ofori-Asenso 2020 | Regional (EU) | Systematic review | HTA | Improving interactions between health technology assessment (HTA) bodies and regulatory agencies | Aligning the activities of HTA/reimbursement bodies and regulatory agencies to inform resource allocation and reimbursement decisions to create the most value for money | A lack of trust and understanding between all stakeholders as well as the lack of an institutional framework for cooperation between HTA bodies and regulatory bodies are major challenges |
| Federici 2021 | Regional (EU) | Qualitative study | Coverage with evidence development (CED) schemes | Cost-effectiveness of new technologies | CED schemes for medical devices collect data on the cost-effectiveness of a health technology and facilitate the uptake of future decisions about its reimbursement, coverage, or recommendations for its use. | -- |
| Bossuyt 2015 | Belgium | Primary study | Public Health Triangulation | Inform decision-making | Public health triangulation is an important tool used to interpret findings from different sources, provide a comprehensive overview of the effectiveness of an intervention (Care Trajectory” program (CT)) and to inform decision-making | Triangulation of data is time consuming |
| Lavrac 2007 | Slovenia | Case study | Data Mining | Improved decision support | Data mining methods including data visualization facilitates data analysis leading to better performance in decision making, as well as improve the effectiveness of developed solutions | -- |
| Martin Fernandez 2021 | France | Realist evaluation study | TC-REG (‘Transfert de Connaissances en REGion’) intervention | Health promotion and disease prevention | The TC-REG intervention aims to synthesize evidence to enhance health promotion and disease prevention focusing on knowledge translation through policy briefs, literature reviews and exploratory qualitative studies |  |
| Haverinen 2022 | Finland | Qualitative study | Digi-HTA | Assessment of digital health technologies | Digi-HTA allows the evaluation of digital health technologies, aiming to determine recommendations to support healthcare decision-makers | -- |
| Kovacs 2022 | Regional | Qualitative study | Coverage with Evidence development (CED) schemes | Evidence-transfer | CED schemes can provide recommendations to lower and reduce decisions uncertainties for reimbursements of medical devices |  |
| Murphy 2022 | Regional | Qualitative study | Evidence briefs for policy (EBPs) | Evidence-informed policy making | EBP provides a systematic approach to contextualizing evidence that is relevant to policy options | How the problem is prioritized, EBP team composition, EBP team leadership, availability of external support, the culture of policy-making in a country, engagement of policymakers |
