# Supplementary material for: Knowledge management tools and mechanisms for evidence-informed decision-making in the WHO European Region: a scoping review
Source: Health Res Policy Syst. 2023 Oct 31;21:113. doi: 10.1186/s12961-023-01058-7 (PMC10619313; doi:10.1186/s12961-023-01058-7)
Supplement: Supplementary file 9 — Additional file 9: Appendix 9. Table of characteristics - Health Reports. [file 12961_2023_1058_MOESM9_ESM.docx]

**Studies on Health Reports (n=5)**

| **Author, Year** | **Country** | **Study design** | **KM tool/Program** | **Policy Outcome(s)** | **Main Results**  **Is the intervention effective overall? (yes/no/inconclusive)** | **Implementation considerations** |
| --- | --- | --- | --- | --- | --- | --- |
| Simovska 2012 | Regional (EU) | Case study | HEPS (Healthy Eating and Physical Activity in Schools) toolkit | Supporting the development of national policies | After completing the HEPS project, an effective Toolkit was developed aiming to support EU countries in developing and implementing national policies on healthy eating and physical activity in schools | Implementation can be faced with several challenges pertaining to the need for a long-term strategy requiring lobbying efforts, and adaptation to current political needs |
| Schoemaker 2019 | The Netherlands | Case study | Public Health Status and Forecasts report (PHSF) | Advise decision-makers | Foresights reports were used to identify four societal challenges for health in The Netherlands, which helped in advising and informing policy makers | -- |
| Hanney 2020 | Regional (EU) | Case study | HEN Report | Strengthening national health research systems | The WHO evidence synthesis published as a HEN report identifies crucial policy approaches (situation analysis, comprehensive strategy, engaging stakeholders, evaluating impacts on health policies and partnership participation) which are considered as tools for researchers to strengthen national health research systems as well as for influencing decision-making | -- |
| Hegger 2016 | The Netherlands | Case study | Health Care Performance Report | Policy-making | Health care performance reporting allows the monitoring of the performance of the Dutch health system, processing information and contributing to knowledge utilization for policy-making | -- |
| Blessings 2017 | Regional (EU) | Report | the European Health Report | Policy-making | Synthesize health information for audience including policy-makers and politicians | -- |
