# Supplementary material for: Knowledge management tools and mechanisms for evidence-informed decision-making in the WHO European Region: a scoping review
Source: Health Res Policy Syst. 2023 Oct 31;21:113. doi: 10.1186/s12961-023-01058-7 (PMC10619313; doi:10.1186/s12961-023-01058-7)
Supplement: Supplementary file 10 — Additional file 10: Appendix 10. Table of characteristics - Policy dialogues. [file 12961_2023_1058_MOESM10_ESM.docx]

**Studies on policy dialogue (n=16)**

| **Author, Year** | **Country** | **Study design** | **KM tool/Program** | **Policy Outcome(s)** | **Main Results**  **Is the intervention effective overall? (yes/no/inconclusive)** | **Implementation considerations** |
| --- | --- | --- | --- | --- | --- | --- |
| Bertram 2018 | Denmark  (sub-national) | uncontrolled before and after | Stakeholder involvement through round table discussions based on evidence assessment of relevant  scientific literature | Use of knowledge in policymaking | In both municipalities, the ability to translate knowledge to local context, the political request and the organizational procedures for use of knowledge increased during the interventions. Most of the changes were diminished at the 12-month follow-up.  Improvement in organizational procedures for the use of research knowledge by policymakers  Increase political request for use of knowledge in policy development. | Ensure sustainability |
| vanKammen 2006 | The Netherland | Case study | Policy dialogue (knowledge brokering process) | Reimbursement decision  Policy formulation | One of the recommendations that would require changes in ministerial policy was followed up instantly, whereas the other recommendation is still under debate.  The Dutch Society of Obstetrics and Gynecology activated the revision of two guidelines.  The patient organization uses the new scientific insights in informing members and the public. | Use of systematic review and HTA results to guide decisions  contributed decisively to the usability of the results.  Timely, concise and consistent use of terminology was more effective than any fragmented approach. |
| Rassenhofer 2013 | Germany | Case study | Critical Incident Reporting System: interim reports and Round Table | Increasing policymaker’s awareness on the issue  Adoption of two federal laws  Agenda-setting and Policy adoption | The government was continuously kept informed through interim reports and through a presentation at each meeting of the Round Table.  After the Round Table was disbanded, the government confirmed its commitment by establishing an authorized representative for matters concerning child sexual abuse, whose tasks included continuing to monitor the implementation of the Round Table’s recommendations.  Two new federal laws were adopted that were based directly on the recommendations of the Round Table; one for strengthening the rights of former victims of sexual abuse, and the other for the current protection of children. | -- |
| Bruen 2020 | Ireland | Qualitative | four policy dialogues (2013–2016) supported by the packaging of research evidence | medical workforce planning | Policy dialogues can support evidence-informed policymaking through improving knowledge exchange and interaction between policy stakeholders and researchers.  the provision of a conducive environment for discussion of timely and relevant summarized evidence; personal contact and ongoing interaction between a range of participants central to health workforce planning in Ireland, and evidence-focused discussion that facilitated a bypassing, of the day-to-day business of negotiation and influence in which policy stakeholders across different organizations often engage | In addition to research evidence, many contextual factors influence medical workforce planning |
| Sienkiewicz, 2020 | Ireland, Portugal and Spain | Case study | CHRODIS PLUS, national policy dialogs | new policies or changes to existing policies and legislation that are capable of tackling major risk factors for chronic disease | CHRODIS PLUS methodology is an effective mechanism to provoke deliberative discussion around chronic disease prevention and management in different countries. | ensure adequate human and financial resources as well as political commitment to accomplish objectives set out during the policy dialogues.  ability of the policy dialogs to facilitate interactions between different sectors—notably between health, finance and environment. |
| Khan 2014 | Switzerland | Case study | Knowledge brokering (sessions to gather information attended by Swiss and foreign experts and other local stakeholders) | Initiatives of a new drug policy | Coalitions of change actors, across stakeholder groups from many professions and politicians on various levels, succeeded in formulating and starting initiatives for a new drug policy and its innovations. | Swiss direct democracy system  Sustained dialogues |
| Jenkins 2009 | Russia (sub-national) | Case study | Policy dialogue as part of a multi-component program to facilitate the integration of mental health into primary care | integration of mental health into primary care | The multi-component program has resulted in sustainable training about common mental disorders, and it has been well integrated with Sverdlovsk’s overall program of health sector reforms. | - |
| Giepmans 2013 | Regional | Case study | a dialogue model of knowledge mobilization of EU-funded research projects | Enhance uptake of research results |  | Targeting the right groups for engagement; understanding their knowledge needs; involving those groups in research co-production; and making full use of all project partners in knowledge mobilization.  building of constructive and sustained working relationships between the researcher and stakeholders  sharing ‘‘tacit knowledge’’ and increasing the understanding of behavior between different stakeholders |
| Turner 2018 | Regional | Narrative/literature review | EXPOsOMICS project “Final Policy Workshop and Stakeholder Consultation” | Policy development | Exposome approach through a policy workshop and stakeholder consultations has a positive impact on risk assessment for common pollutants and linking exposure to diseases and on preventative and regulatory actions | Communicate research results in a lay language to non-scientific audience |
| Gouveia 2008 | Portugal and EU | Case study | Round Table discussion/Conference | Developing the EU Health Strategy | Discussions during the round table have helped in developing recommendations for cancer plans, registration and screening, leading to the development of the EU Health Strategy for cancer control | -- |
| Ditchburn 2016 | United Kingdom | Delphi techniques | Cumbria Rural Health Forum | Cumbria Strategy for Digital Technologies in Health and Social Care | Adopt recommendations from the forum to influence and advocate the implementation of evidence-based best practices for digital health and social care delivery in Cumbria | -- |
| Muszbek 2007 | Hungary | Case study | Open Society Institute Conferences | Integrating palliative care into the Hungarian National Cancer Control Program | The conference facilitated and indorsed the change of palliative care financing, doubling the palliative care capacity and integrating it in the national program on cancer control | -- |
| Patera 2013 | the Netherlands, the United Kingdom and  Norway | Qualitative study | Forums or interaction | Policy making for health gains | Research commissioning Institutions influence research-stakeholder interaction to encourage relevance and usability of research evidence to support policy making | -- |
| deBruin 2018 | Europe | Case study | SUSTAIN Project (suitable tailored integrated care for older people in Europe) | Improve integrated care | The SUSTAIN project enhances collaboration with local stakeholders to support improvements in integrated care for older people as well as adapt these improvements into different regions across Europe | -- |
| O'Connor 2021 | Regional (EU) | Mixed method | Knowledge Translation Planning | Enhanced research visibility for stakeholders | Having a knowledge translation plan allows the dissemination of findings from research to stakeholders, leading to better research visibility and more effective translation of evidence for better policy outcomes | -- |
| vanKammen 2006 | The Netherlands | Narrative/literature review | Knowledge Brokering: REACH-Policy Initiative | Rational policy decision-making | The REACH-policy initiative links the use of research to evidence-based policy making through knowledge brokering | -- |
| Storm 2015 | The Netherlands | Case study | Policy dialogue and district health profiles | integrated planning of health activities | Stepwise approach combining use of integrated district health profiles and policy dialogues leads to integrated health plans or activities aimed at improving the health of the local population | -- |
