# Supplementary material for: Knowledge management tools and mechanisms for evidence-informed decision-making in the WHO European Region: a scoping review
Source: Health Res Policy Syst. 2023 Oct 31;21:113. doi: 10.1186/s12961-023-01058-7 (PMC10619313; doi:10.1186/s12961-023-01058-7)
Supplement: Supplementary file 11 — Additional file 11: Appendix 11. Table of characteristics - Evidence networks. [file 12961_2023_1058_MOESM11_ESM.docx]

**Evidence Networks (n=31)**

| **Author, Year** | **Country** | **Study design** | **KM tool/Program** | **Policy Outcome(s)** | **Main Results**  **Is the intervention effective overall? (yes/no/inconclusive)** | **Implementation considerations** |
| --- | --- | --- | --- | --- | --- | --- |
| Jansen 2022 | Regional | Case study | Health Technology Assessment bodies (HTABs) | Decision making | HTABs allow the dissemination of evidence across stakeholders for decision making | **--** |
| Renzella 2018 | Europe | Technical report | Health Evidence Network | Policy formulation | To support decision-makers in shaping context-specific diet and nutrition policies | **--** |
| Spitters 2017 | multinational | System analysis | Stakeholder network | Decision making | Support decision makers across key public health issues | **--** |
| Spitters 2018 | Multinational | Mixed method (qualitative and quantitative) | In2Action: stakeholder networks | Real-life work in local health policymaking | The policy game In2Action provided important learning experiences for the participants, which added value to real-life work in local health policymaking | **--** |
| Vogler 2014 | Europe | Qualitative study | The Austrian Health Institute and the World Health Organization (WHO) Regional Office for Europe  A network of competent authorities to improve information | Pharmaceutical policies | To facilitate access to specific country information Launch of more than 200 ‘PPRI network queries’ by policy makers to support pharmaceutical policies | **--** |
| Bartonova 2012 | Europe | Case study | HENVINET project  network | Bridge the communication gap between science and society. | The HENVINET approach lead to the establishment of Interactive web-based tools to enhance methods for knowledge evaluation, and use these methods to formulate policy advice. | **--** |
| Lester 2020 | Europe | Mixed Method | Evidence-informed Policy Network (EVIPNet) Europe | Bridging the research-policy-practice gap and enable the effective | Formulation of policy advice | Barriers are the lack of relevance and availability of research, lack of skilled policymakers, insufficient institutional research capacity, inadequate dissemination of evidence, time constraints to the use of evidence, lack of funding, limited resources or high costs, and a lack of priority on the policy agenda.  Facilitators for use include improved dissemination and access to research, administrative support, training of personnel, the fostering of research co-production as well as research led by people embedded in the contexts in which the results can be used. |
| Mihalicza 2018 | Hungary | Qualitative study | EVIPNet Europe | Bridging the research-policy-practice gap and enable the effective | Evidence-informed policy | Barriers and facilitators for evidence-informed health policy practice  Barriers to use include opposing interests, time constraints to the use of evidence, lack of funding, limited resources or high costs, and a lack of priority on the policy agenda.  Facilitators for use include availability of a sectoral strategy, good quality professionals, strong political authority of the government in decision making. |
| Luukkainen 2006 | Finland | Qualitative study | Finnish National Healthy Cities Network  Knowledge transfer | Agenda setting and policy formulation | Develop and implement health promotion policies, support national health promotion policy and evaluation | -- |
| De Haas 2017 | Netherlands | Mixed Method (Narrative and qualitative) | Share-Net | Evidence informed policy | -- | Suggestions for improving the flow of knowledge include creation of a visible and concrete demand, production of a joint knowledge agenda, and development of a system for learning and knowledge sharing |
| Butler, 2013 | Denmark, Norway, Sweden, and United Kingdom | Case study | Research collaboration: The International Cancer Benchmarking Partnership  Knowledge transfer | Evidence informed cancer policies | Contribute to policy development in the UK including the setting of levels of Ambition for the NHS Outcomes framework  In Wales, England and Victoria lack of staging and treatment information has led to policies to improve the quality of registry recording of stage | **--** |
| McAteer 2019 | UK | Opinion pieces/editorials/commentaries | Scottish Collaboration for Public Health Research and Policy | Advise during the SARS-CoV-2 pandemic. | Assessment of the evaluability of any public health program or policy that is either implemented or being considered for implementation in the future | **---** |
| Gombos 2021 | Hungary | Case study | "KETLAK"  Translational Action and Research Group against Coronavirus | Agenda setting and policy formulation | Research activity and participation in decision-making | **--** |
| Hoeijmakers, 2013 | Netherlands | Qualitative study | Academic Collaborative Centres for Public Health | Evidence based policy | Evidence base of public health policy and practice aiming at improvement of the health of the region’s 600,000 inhabitants.  Public health themes covered by the ACCL have included health promotion, preventive youth care and health policy | **--** |
| Wehrens 2014 | Netherlands | Case study | Academic Collaborative  Centres for Public Health | Agenda setting | Development of policy relevant scenarios, in which scientific elements and policy elements were closely linked to the policy program.  Collaborations at the research and academic levels act as knowledge management tools and form an evidence base for public health policy and practice | **--** |
| Stark 2013 | Scotland | Mixed method (Literature review and Survey) | Knowledge Transfer Partnership (KTP). | Implementation of national care standards, including diagnostic standards | Implementation of national care standards, including diagnostic standards, and review of dementia services | **--** |
| Sell, 2021 | Germany | Narrative/literature review | Expert committee convention | Policy making | Study investigated the disciplinary composition, gender representation and the transparency related to the convening of these bodies, work processes and the accessibility of results. | Women made up only around quarter of the members. Biomedical disciplines such as virology, hygiene, medicine, and biology were the most represented. In eleven out of twenty-one expert committees, members were known by name. Members of the remaining ten committees who were not known by name were more commonly practitioners or came from affected populations. These committees covered different thematic areas such as school and day-care, civil participation, medicine and care, economic topics. |
| Tiessen, J., et al. (2011). | Europe | Case study | DG SANCO Data Management Practices  The European Commission Health and Consumer Protection Directorate-General (DG SANCO) | -- | -- | Perceived challenges were the limited availability of data and uncertainty about potential data sources.  High-level recommendations stress on the importance of a shift in organization culture |
| Aro, 2016 | Denmark, The Nehterlands, Finland, Romania, Italy, UK, Canada | Case study | REsearch into POlicy to enhance Physical Activity (REPOPA). | Physical activity policy-making | Extensive research evidence would be used and frequent collaboration between investigators and policy makers would increase evidence-informed policy making for physical activity. | -- |
| vanderGraaf, 2019 | England | Commentary | Knowledge brokering service: AskFuse | Increase the uptake of research evidence in policy making | Brokering access to academic expertise and research for policy-makers to make evidence-based decisions | Collaborations are sometimes resisted by policymakers |
| Loblova 2018 | EU | Case study | Epistemic communities in policy-making | Shaping policy-makers preferences | Epistemic communities achieve their policy goal through spreading views and arguments, then gaining access to decision-makers and finally convincing decision-makers of their policy solution. In Poland, epistemic communities were able to convince the Minister that HTA was the response to urgent problems | -- |
| Kristensen 2009 | Regional | Case study | EUnetHTA | Reimbursement decision | European network for Health Technology Assessment HTA (EUnetHTA) aims at examining the HTA process and its link to policy making | **--** |
| Kristensen 2006 | Regional | Case study | European Network for Health Technology Assessment project (EUnetHTA project) | Support policy decisions | Connecting public national HTA agencies, research institutions, and health ministries to enable an effective exchange of information and lend support to policy decisions by Member States | **--** |
| Kristensen 2009 | Europe | Case study | EUnetHTA | Agenda setting and policy formulation | Structure prioritization for HTA and provide healthcare decision makers with policy relevant information on new and emerging technologies  Develop and implement effective tools to transfer HTA results into applicable health policy advice in the Member States and EU | **--** |
| Malterud 2016 | Norway | Case study | Norwegian Knowledge Centre for the Health Services (NOKC) | Evidence-based policy making | Adopting evidence-based medicine in the NOKC neglects the concept on context in evidence-based policy making highlighting the fact that decision making process require more than just knowledge translation | -- |
| Huibers 2014 | Europe | Narrative/literature review | European research network for out-of-hours primary health care (EurOOHnet), | Policy making in OOH care | The EurOOHnet shares research allowing knowledge transfer for policy-makers, facilitating high-quality OOH care |  |
| Perkins 2016 | Wales | Primary study | The Life Science Exchange® project | Sector-specific policies through knowledge exchange and collaborations | The Life Science Exchange project facilitated interactions and collaborations among different clinical, academic and governmental entities, exchanging information and improving skills | -- |
| Keune, 2012 | Regional (EU) | Case study | HENVINET | Informed policy making | The FP6 EU HENVINET aims at disseminating knowledge on environmental and health issues (e.g. the influence of environment health stressors on cancer induction) based on research, for informed policy making | -- |
| David 2020 | Regional | Case study | Health information systems implemented by BRIDGE Health (Bridging Information and Data Generation for Evidence-based Health policy and Research) and HBM4EU (European human biomonitoring initiative) |  | BRIDGE Health had a special task on the transferability of HI and its data for policy.  Combining HBM and HES, (b) indicator development and (c) linking data repositories. | This data is mostly aggregated, and only poorly interlinked with  each other. In addition, there are strong inequalities in data availability and quality, and a lack of appropriate  means and formats for data transfer exists.  Harmonization of data, data transfer, and data protection rules are  required. |
| Smolders 2008 | Europe | Case study | Human biomonitoring data | Policy evaluation | Evaluate the efficacity of existing regulations such as tobacco laws specifically monitoring cotinine in the urine of children, which is a tracer of exposure to passive smoking and allows the assessment of whether the implementation of these laws in different countries has been successful in terms of reducing exposure to a vulnerable population. | *Human biomonitoring is a unique tool to evaluate the efficacy of the program, provided that validated biomarkers for the chemicals under consideration are available.* |
| Haneef 2021 | Europe | Mixed method | Burden of Disease (BoD) approach | Health and policy improvement | Burden of Disease studies allow the assessment and quantification of the disease facilitating the transformation of standards measurements (DALYs, YLL, YLD) to integrative measures in policy | -- |
