# Supplementary material for: Knowledge management tools and mechanisms for evidence-informed decision-making in the WHO European Region: a scoping review
Source: Health Res Policy Syst. 2023 Oct 31;21:113. doi: 10.1186/s12961-023-01058-7 (PMC10619313; doi:10.1186/s12961-023-01058-7)
Supplement: Supplementary file 12 — Additional file 12: Appendix 12. Table of characteristics - Community engagement. [file 12961_2023_1058_MOESM12_ESM.docx]

**Studies on Community Engagement (n=5)**

| **Author, Year** | **Country** | **Study design** | **KM tool/Program** | **Policy Outcome(s)** | **Main Results**  **Is the intervention effective overall? (yes/no/inconclusive)** | **Implementation considerations** |
| --- | --- | --- | --- | --- | --- | --- |
| Hankivsky 2017 | Ukraine | Qualitative | Community consultation | Health reform | Provides policy-makers with additional evidence to ensure that health reforms would include a focus not only on health system changes but also social determinants of health. | - |
| King 2009 | Scotland  (sub-national) | Qualitative | Community consultation | health and social care service provision for older people | Inform policy-makers about changing or improving service delivery | - |
| Weiler 2013 | England  (sub-national) | Case study | Community consultation through BJSM blog | National Health Service strategies | Including physical activity as a priority in the revised strategy | - |
| Reynolds 2020 | England | Qualitative | Community engagement | National policies on controlling alcohol availability | Community engagement can lead to policy change (licensing policy) | *“Four engagement modalities: as part of statutory consultation processes (particularly licensing); as part of the development of new policies (such as CIPs); through representative structures (such as committees for the local economy); and arising in a more ad hoc way through other activities (such as a student union campaign around sexual harassment)”* |
| Payne 2017 | United Kingdom | Opinion pieces/editorials/commentaries | Pathways2Wellbeing | Engaging and informing the community for potential impact and action | Knowledge transfer using the Pathways2Wellbeing website assisted in providing insights to the community on how engagement with stakeholders can result in impact on health service policy | -- |
