# Supplementary material for: Knowledge management tools and mechanisms for evidence-informed decision-making in the WHO European Region: a scoping review
Source: Health Res Policy Syst. 2023 Oct 31;21:113. doi: 10.1186/s12961-023-01058-7 (PMC10619313; doi:10.1186/s12961-023-01058-7)
Supplement: Supplementary file 13 — Additional file 13: Appendix 13. Table of characteristics - Decision Support Tools. [file 12961_2023_1058_MOESM13_ESM.docx]

**Studies on Decision Support Tools (n=5)**

| **Author, Year** | **Country** | **Study design** | **KM tool/Program** | **Policy Outcome(s)** | **Main Results**  **Is the intervention effective overall? (yes/no/inconclusive)** | **Implementation considerations** |
| --- | --- | --- | --- | --- | --- | --- |
| Cresswell 2019 | United Kingdom | Qualitative study | NHS Scotland DST Platform | Policy making | The use of Decision support tools facilitates the integration of preventive care, patient-engagement and shared decision-making through making information readily available, in turn supporting policy making | -- |
| Liu 2012 | Region (EU) | observational study | The HENVINET DST MDB (Health and Environment) | Balanced decisions based on all stressors-based DSTs | DSTs make information available on different stressors, allowing policy-makers to take balanced decisions | the need to develop DSTs for more than one decision-making area since a lot of DSTs are focused on only one stressor, neglecting other important diseases |
| Martensen 2019 | Belgium | Case Study | Decision Support System | Implementing evidence-based strategies for road safety | A decision support innovative system enables policy-makers and stakeholders to select and implement the most appropriate strategies to enhance road safety by reducing casualties of all road user types | -- |
| Deloly 2021 | France | Case Study | Cities Rapid Assessment Framework for Transformation (CRAFT) policy decision-support tool | Decision making | The implementation of the CRAFT tool has resulted in the building of trust among stakeholders, raise awareness of health issues among decision-makers and, above all, improved collaboration. | -- |
| Chung 208 | Spain | Case Study | the SOMNet, combined with the EbCA | Mental health planning | *“The use of the SOMNet approach contributes to the demonstration of DSS for mental health planning in practice.”* | -- |
